# Filaments Production and Fused Deposition Modelling of ABS/Carbon Nanotubes Composites

**DOI:** 10.3390/nano8010049

**Published:** 2018-01-18

**Authors:** Sithiprumnea Dul, Luca Fambri, Alessandro Pegoretti

**Affiliations:** Department of Industrial Engineering and INSTM Research Unit, University of Trento, Via Sommarive 9, 38123 Trento, Italy; sithiprumnea.dul@unitn.it (S.D.); luca.fambri@unitn.it (L.F.)

**Keywords:** conductive composites, carbon nanotubes, fused deposition modelling, mechanical properties

## Abstract

Composite acrylonitrile–butadiene–styrene (ABS)/carbon nanotubes (CNT) filaments at 1, 2, 4, 6 and 8 wt %, suitable for fused deposition modelling (FDM) were obtained by using a completely solvent-free process based on direct melt compounding and extrusion. The optimal CNT content in the filaments for FDM was found to be 6 wt %; for this composite, a detailed investigation of the thermal, mechanical and electrical properties was performed. Presence of CNT in ABS filaments and 3D-printed parts resulted in a significant enhancement of the tensile modulus and strength, accompanied by a reduction of the elongation at break. As documented by dynamic mechanical thermal analysis, the stiffening effect of CNTs in ABS is particularly pronounced at high temperatures. Besides, the presence of CNT in 3D-printed parts accounts for better creep and thermal dimensional stabilities of 3D-printed parts, accompanied by a reduction of the coefficient of thermal expansion). 3D-printed nanocomposite samples with 6 wt % of CNT exhibited a good electrical conductivity, even if lower than pristine composite filaments.

## 1. Introduction

The development of nanocomposite materials for specific types of additional manufacturing has recently attracted remarkable interest because incorporated nanoparticles offer the potential to enhance various properties of 3D-printed parts [1,2,3]. In particular, filaments for fused deposition modelling (FDM)—which is a widely used 3D-printing technology—could be improved by the addition of nanofillers. In fact, the dispersion of conductive nanoparticles in a polymer matrix makes it possible to produce 3D-printed components for various applications such as electronic sensors [4,5,6], cases with good electromagnetic interference (EMI) shielding performances [7], circuits [8] and microbatteries [9].

To date, various conductive nanoparticles have been used in 3D printing, such as carbon black (CB) [4,10], graphene oxide (GO) [11,12], reduced graphene oxide (r-GO) [8], graphene [13,14] and carbon nanotubes [5,13,15,16,17]. However, very few studies have been focused on the production of nanocomposite filament feedstock for FDM. For example Zhang et al. [8] reported the resistivity of composite filaments with a diameter of 1.75 mm of r-GO/Polylactic acid (PLA) of 0.21 Ω·cm (6 wt % r-GO), along with the superior mechanical properties of FDM parts. Zhang et al. [10] reported the effect of 15 wt % of CB on the resistivity of composite ABS feedstock filaments (about 2900 Ω·cm) and characterized the resistivity of 3D-printed parts under various FDM parameters. Wu et al. dispersed up to 3 wt % multi-walled carbon nanotubes (MWCNTs) in polyhydroxyalkanoate to produce feedstock filaments but the resistivity of filaments have not been reported [17]. Wei et al. [11] were able to produce 3D printed parts with 5.6 wt % of GO in ABS matrix but they did not investigate electrical or mechanical properties. Gnanasekaran et al. [13] reported on the 3D printing with polymer nanocomposites consisting of CNT- and graphene-based polybutylene terephthalate, finding that 3D-printed objects filled with CNT have better conductive and mechanical properties and better performance than those filled with graphene. In our recent study [14] we have used for the first time acrylonitrile–butadiene–styrene (ABS) matrix filled with 4 wt % graphene nanoplatelets (xGnP); the composite filaments were obtained by a solvent-free process consisting of melt compounding and extrusion.

In a previous paper [18], the main focus was the possibility of dispersing CNT in ABS by using a commercial masterbatch of ABS/CNT for the production of filaments with a non-standard diameter of 1.4 mm. Six wt % of CNT was found to be an optimal fraction for the production of composite filaments.

In this study, we have investigated the possibility to directly disperse CNT in ABS matrix in order to produce the ABS/CNT filaments suitable for the FDM process with a standard diameter of about 1.7 mm. Nanocomposite filaments were manufactured by using common industrial processing techniques such as internal mixer and twin-screw extruder to compound polymer pellets (without additives) with CNT nanofiller. Relatively higher viscosity ABS matrix and lower processing temperatures with respect to the previous paper have been properly selected in order to increase the processing shear stresses and to improve/facilitate CNT dispersion. Extensive thermal, mechanical and electrical characterization of the obtained filaments was carried out. Afterwards, selected filaments were used to feed a high-temperature FDM 3D printer to specify the effects of CNT on the properties 3D-printed components along various build orientations.

## 2. Materials and Methods

### 2.1. Materials

The acrylonitrile-butadiene-styrene (ABS) polymer (tradename Sinkral^®^F322) used in this study was kindly provided by Versalis S.p.A. (Mantova, Italy). According to producer’s technical data sheet, the polymer is characterized by a density of 1.04 g/cm^3^ and a melt volume rate of 14 cm^3^/10 min (@220 °C/10 kg) [19]. Before processing, ABS chips were dried under vacuum at 80 °C for at least 2 h.

Multi-walled carbon nanotubes (CNTs) (tradename NC7000^TM^) were provided by Nanocyl S.A. Sambreville, Belgium). The technical data sheet reports an average length of 1.5 μm, a diameter of 9.5 nm and a surface area of 250–300 m^2^/g [20].

### 2.2. Materials Processing and Sample Preparations

#### 2.2.1. Filament Extrusion

In order to maintain a proper distribution of nanofiller in the matrix, various amounts (1, 2, 4, 6 and 8 wt %) of CNTs were first melt blending with ABS matrix through a Thermo-Haake Polylab Rheomix counter-rotating internal mixer (Thermo Haake, Karlsruhe, Germany) at a temperature of 190 °C and rotor speed of 90 rpm for 15 min. The neat ABS was also processed under the same conditions. The resulting material was granulated in a Piovan grinder Model RN 166 (Piovan, S. Maria di Sala VE, Italy). Then the batches were used to feed a Thermo Haake PTW16 intermeshing, co-rotating twin screw extruder produced by Thermo Haake, Karlsruhe, Germany (screw diameter *D* = 16 mm; *L*/*D* ratio = 25, where *L* is screw length; rod die diameter 1.80 mm). The temperature profile was set as *T*_1_ = 180 °C, *T*_2_ = 205 °C, *T*_3_ = 210 °C, *T*_4_ = 215 °C and *T*_5_ = 220 °C. Filaments with a nominal diameter of about 1.70 mm were collected by using a take-up unit Thermo Electron Type 002-5341 (Thermo Haake, Karlsruhe, Germany) at constant collection rate. The main parameters adopted for the filament production are summarized in Table 1.

ABS denotes filament of pure matrix; whereas CNT6 indicates composite filament with 6 wt % of CNTs.

#### 2.2.2. 3D-Printed Fibers

3D-printed fibers were prepared, starting from extruded filament by using a prototype of a 3D printer for high temperature processing, Sharebot HT Next Generation desktop (Sharebot NG, Nibionno, LC, Italy). through a nozzle diameter of 0.40 mm at temperature of 250 °C or 280 °C for ABS or composites respectively. Fibers of about 100 cm length with a diameter of 0.50–0.65 mm were freely extruded at an extrusion speed of 40 mm/s for mechanical and electrical testing.

#### 2.2.3. 3D-Printed Samples Preparation

**Figure 1:**
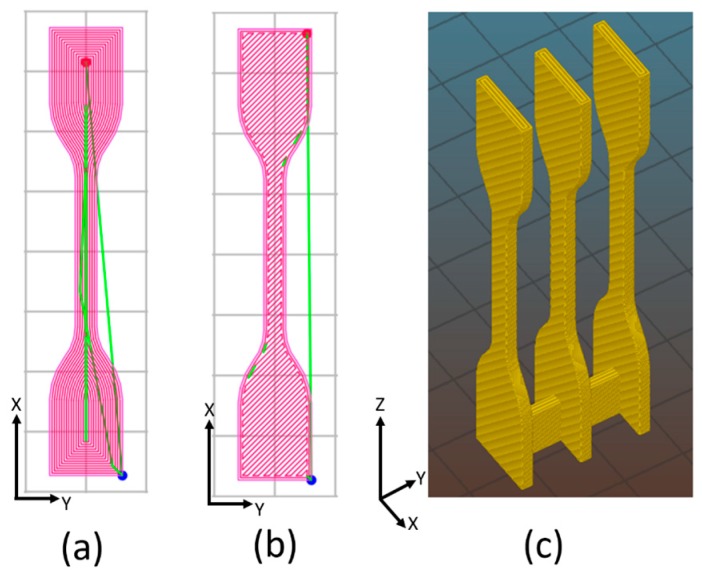
Schematic of 3D-printed dumbbell: (**a**) horizontal concentric (HC); (**b**) horizontal 45°angle (H45) and (**c**) vertical concentric (VC).

3D-printed specimens were manufactured by feeding the 3D printer described in Section 2.2.2 with the filaments obtained as described in Section 2.2.1. As schematically depicted in Figure 1, dumbbell and parallelepiped specimens were built-up along different build orientation, i.e., horizontal concentric (HC (a)), horizontal 45° angle (H45 (b)) and vertical concentric (VC(c)). All samples were printed according to the following printing parameters: object infill 100%; no raft; nozzle diameter 0.40 mm; nozzle temperature 250 °C and 280 °C for neat ABS and nanocomposite respectively; bed temperature 110 °C, layer height 0.20 mm; infill speed 40 mm/s; raster angle and printing speed are reported in Table 2.

Each specimen was individually printed, with the exception of VC samples for tensile, dynamic mechanical thermal analysis (DMTA) and creep tests (three specimens per printing session were simultaneously grown). 3D-printed samples were denoted indicating the material composition followed by build orientation. For example, CNT6-HC indicates the 3D-printed composite specimen with 6 wt % of CNT at HC build orientation.

### 2.3. Testing Techniques

#### 2.3.1. Density Measurement

The density of the carbon nanotube was determined by using a Micromeritics^®^Accupyc 1330 helium pycnometry (Micromeritics, Norcross, GA, USA) at 23 °C within 300 measurements in a 10 cm^3^ chamber; the average values and standard deviation of the last 200 measurements are reported (see details in Supplementary Materials). 

Density measurement of bulk composite filaments was performed according to the standard ASTM D792-13. Moreover, the theoretical density and the voids content in nanocomposites were evaluated through the rule of mixture, as detailed in Supplementary Materials Section 2 (Equations (S1)–(S3)). 

Linear density of the filament and the fiber was expressed in tex, according to ASTM D681-07, as the weight in grams of 1000 m of product and it was determined by weighting specimens of at least 90 mm in length. The results are the average of five measurements.

#### 2.3.2. Melt Flow Index

The melt flow index (MFI) analysis of extruded filaments was carried out according to the ASTM D 1238 standard (procedure A), through a Kayeness Co. model 4003DE capillary rheometer (Morgantown, PA, USA) (barrel length of 162 mm and barrel diameter of 9.55 mm; die length of 8.000 mm and die diameter of 2.096 mm). About 5 g of chopped filament were tested at a temperature of 250 °C with an applied load of 10 kg, after the pre-heat and compact time of about 5 min. Pure ABS pellets were also tested at 220 °C and 10 kg. The results represent the average of at least five measurements (standard deviation is reported).

#### 2.3.3. Scanning Electron Microscopy

Morphology of nanocomposites was studied by using a Carl Zeiss AG Supra 40 field emission scanning electron microscope (FESEM) (Carl Zeiss AG, Oberkochen, Germany). Specimens were fractured in liquid nitrogen and the fracture surfaces were observed at an acceleration voltage of 4 kV. Representative micrographs of filaments and 3D-printed dumbbell at 6 and 8 wt % of CNT were selected.

#### 2.3.4. Thermogravimetric Analysis (TGA)

Thermal degradation was investigated through a Q5000 IR thermogravimetric analyzer (TA Instruments-Waters LLC, New Castle, DE, USA). The samples had a mass of about 10 mg and tests were performed in an air flow of 15 mL/min from 30 °C to 700 °C at a rate of 10 °C/min. The onset temperature of degradation (*T*_onset_) was defined by the intersection point of the two tangent lines and the maximum temperatures (*T*_max_) correspond to the maximum of the first derivative of weight loss. The residue at 475 °C, 575 °C and 700 °C were also reported in order to evaluate the content of CNT (*C*_CNT_) according to the equation:

*C*_CNT_ = *R*_comp_ − *R*_ABS_ (1)

where *R*_comp_ and *R*_ABS_ are the residue of composite and ABS respectively at the same temperature.

#### 2.3.5. Differential Scanning Calorimetry (DSC)

DSC analyses were performed by a Mettler DSC 30 calorimeter (Mettler Toledo, Columbus, OH, USA) on samples with a mass of about 10 mg under a nitrogen flow of 100 mL/min. The samples were tested under heating-cooling-heating cycle from 30 °C to 260 °C at a rate of ±10 °C /min. Glass transition temperature (*T*_g_) of styrene–acrylonitrile copolymer (SAN) phase was measured as the inflection point of the thermograms.

#### 2.3.6. Quasi-Static Tensile Test

Uniaxial tensile test on filaments and 3D-printed samples were carried out at room temperature through an Instron^®^ 5969 electromechanical tester (Norwood, MA, USA) equipped with a load cell of 50 kN. Yield and fracture properties were evaluated at a crosshead speed of 10 mm/min as an average value of at least three replicates. Filaments specimens had a length of 150 mm, a gauge length of 100 mm and a diameter of 1.70 mm. 3D-printed specimens (HC, H45 and VC) had a dumbbell geometry according to ISO 527 type 5A with a gauge length of 25 mm, a width of 4 mm and a thickness of 2 mm.

Tensile properties of 3D-printed fibers were determined by using an Instron^®^ 5969 electromechanical tester equipped with a load cell of 100 N. Fiber specimens with a diameter between 500–650 micron and a gauge length of 20 mm were tested at a cross-head speed of 2 mm/min.

Elastic modulus of 3D-printed samples was determined at a cross-head speed of 1 mm/min by an electrical extensometer Instron^®^ model 2620-601 (Norwood, MA, USA) with a gage length of 12.5 mm. Elastic modulus of filaments with a gage length of 100 mm and fiber with a gage length of 20 mm was tested at 10 mm/min and 2 mm/min, respectively, taking the system compliance into account. According to ISO 527 standard, the elastic modulus was determined as a secant value between strain levels of 0.05% and 0.25%.

#### 2.3.7. Creep Test

Creep test was performed using a TA Instruments DMA Q800 (TA Instruments-Waters LLC, New Castle, DE, USA) at 30 °C up to 3600 s under a constant stress of 3.9 MPa on cylindrical extruded filament specimens and of 3 MPa on a 3D-printed rectangular sample with a width of 4 mm and a thickness of 1 mm. For all specimens, an overall the length of 25 mm was used and the adopted gauge length of all samples was 11.5 mm.

#### 2.3.8. Dynamic Mechanical Thermal Analysis

Dynamic mechanical thermal analysis (DMTA) tests were performed under tensile mode by a TA Instruments DMA Q800 device (TA Instruments-Waters LLC, New Castle, DE, USA). 3D-printed specimens with a width of 4 mm and a thickness 2 mm were used. All specimens had an overall length of 5 mm and a gauge length of 11.5 mm. Tests were performed from 100 °C to 150 °C at a heating rate of 3 °C/min applying a dynamic maximum strain of 0.05% at a frequency of 1 Hz. Storage modulus (*E*’), loss modulus (*E*’’) and loss tangent (tanδ) as a function of the temperature were reported. According to the manufacture data sheet, the precision on storage modulus is ±1% [21].

In order to evaluate the stiffness effect of the filler in nanocomposites above *T*_g_, the reduction of the main transition *R* has been defined in Equation (2) as the ratio of storage modulus above *T*_g_ (i.e., at 130 °C) and storage modulus below *T*_g_ (i.e., at 90 °C), after modification of the equation S “intensity of transition” previously defined [22].

(2) $$R=\frac{{E^{\prime }}_{130{}^{\circ}C}}{{E^{\prime }}_{90{}^{\circ}C}}$$

Moreover, following Equation (2), a *F*-factor has been defined as in Equation (3) as the relative *R* ratio between composite and matrix and it is formally derived from the inverse of *C*-factor reported in the literature [23,24]:

(3) $$F=\frac{R_{\mathrm{composite}}}{R_{\mathrm{matrix}}}=\frac{{\left({E^{\prime }}_{130{}^{\circ}C}/{E^{\prime }}_{90{}^{\circ}C}\right)}_{\mathrm{composite}}}{{\left({E^{\prime }}_{130{}^{\circ}C}/{E^{\prime }}_{90{}^{\circ}C}\right)}_{\mathrm{matrix}}}$$

The coefficient of linear thermal expansion (CLTE) in four intervals below *T*_g_ (i.e., −50~−20 °C; 20~50 °C; 70~90 °C, 108~113 °C) and a coefficient of linear thermal deformation (CLTD) above *T*_g_ in the range 130~150 °C were determined according to Equation (4). CLTE or CLTD were obtained by linear-fitting the experimental data of thermal strain as a function of temperature.

(4) $$CLTE\;or\;CLTD=\frac{\Delta L/L_{0}}{\Delta T_{0}}$$

where *L*_0_ and Δ*L* are the initial specimen gauge length and the length variation and Δ*T*_0_ is the selected temperature interval.

#### 2.3.9. Electrical Resistivity Test

The test was carried out following ASTM D4496-04 standard for moderately conductive materials under a four-point contact configuration. Each specimen was subjected to a voltage in the range 2–24 V by using a direct current (DC) power supply IPS303DD produced by ISO-TECH (Milan, Italy) while the current flow across it between external electrodes was measured by using an ISO-TECH IDM 67 Pocket Multimeter electrometer (ISO-TECH, Milan, Italy). Composite filaments, fibers and 3D-printed samples (cross-section 6 mm × 2 mm) with a length of 25 mm were tested at 23 ± 1 °C at different voltage; resistivity values represent the average of at least three specimens. Due to the rough surface of 3D-printed samples, a conductive silver paint was applied between the specimen surfaces at the contact electrodes in order to reduce contact resistance. The electrical volume resistivity of the samples was evaluated as follows:

(5) $$\rho =R\cdot \frac{A}{L}$$

where *R* is the electrical resistance, *A* is the is the cross-section of the specimen and *L* is the distance between the internal electrodes (i.e., 3.69 mm).

The heating of a specimen generated by a current flow is known as resistive heating and it is described by the Joule’s law. Surface temperature evolution induced by Joule’s effect upon different applied voltages was measured by using a Flir E6 thermographic camera (FLIR System, Wilsonville, OR, USA). The voltages were applied by a DC power supply (IPS 303DD produced by ISO-TECH), while the samples were fixed with two metal clips with an external distance of 30 mm. In these tests, specimen length was 50 mm with different cross-sections for filaments (about 2.3 mm^2^) and 3D-printed (12.0 mm^2^) specimens. The surface temperature values were recorded for 120 s under the application of voltages of 12 V and 24 V.

## 3. Results and Discussion

The first step of composite preparation was performed following compounding procedure with the direct mixing of filler and polymeric matrix as previously reported in [14,25]. For the purpose to increase the processing shear stresses during CNT dispersion, an ABS matrix with MFI of 14.8 ± 1.0 g/cm^3^ (220 °C and 10 kg) was properly selected, with viscosity higher than ABS with MF of 23 g/cm^3^ (220 °C and 10 kg) previously utilized for the production CNT composite from master-batch [18]. Moreover, in the second step of filament extrusion, lower processing temperatures, 220 °C instead of 240 °C [18], were set in order to furtherly improve dispersion under high shear stresses.

### 3.1. Filament Extrusion and Melt Flow Index

The filament of neat ABS and of ABS/CNT composites were extruded with an orientation factor of about 1.0 at 220 °C, as evaluated by the ratio between the cross-sectional area of the extruder die hole (*S*_DE_) and the cross-sectional area of the obtained filament (*S*_F_) according to Equation (6)

*OF*_E_ = *S*_DE_/*S*_F_ (6)

Moreover, the orientation factor of fiber produced by 3D-printer as the cross-sectional area of filament (*S*_F_) and the cross-sectional area of the obtained fiber (*S*_f_), according to Equation (7)

*OF*_3D_ = *S*_F_/*S*_f_ (7)

The orientation factor is higher in the fiber (produced at 250 °C for ABS and at 280 °C for CNT6) than filament obtained at 220 °C due to the processing conditions. The higher the CNT content, the higher the orientation factor of fibers. Moreover, it is important to observe that linear density of fiber is progressively decreasing with CNT content, as shown in Table 3.

This result could be explained by considering that the final diameter of fiber is decreasing with the nanofiller content (see detail in paragraph 3.6). Consequently, the free flow of the fibers from die of 3D-printer was used to evaluate the die-swelling (*DS*), according to Equation (8), where *S*_f_ is the cross-sectional area of fibers and *S*_DP_ is nozzle section of 3D-printer.

*DS* = *S*_F_/*S*_DP_ (8)

Table 3 shows that die-swelling of investigated composites is significantly reduced as the CNTs fraction increases; in particular, at 6 and 8 wt % of CNTs, die swelling in fiber is almost completely suppressed.

Moreover, the total orientation factor in fiber *OF*_T_ could be calculated combining Equations (6) and (7), as shown in Equation (9):

*OF*_T_ = *S*_DE_/*S*_f_ (9)

The total orientation factor in the fiber increased with CNT content in direct dependence on the first step of filament production at 220 °C and the subsequent extrusion from 3D printer at 250 °C (for ABS) or 280 °C (for nanocomposite), that is the most effective step. This cumulative effect could be a useful parameter for evaluating the processability of the various filaments.

**Figure 2:**
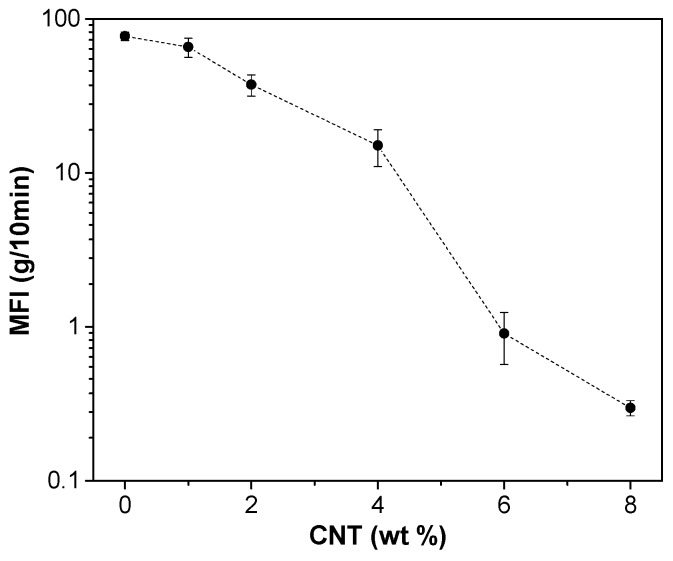
Melt flow index of ABS nanocomposites as a function of CNT fraction at 250 °C with applied load 10 kg.

The effect of CNT on the melt flow index (MFI) of extruded ABS filaments was also investigated. Figure 2 shows a strong decrease of MFI with the carbon nanotubes content, due to the increasing viscosity induced by the formation of a nanofiller network. This effect is also documented by a significant increase in the torque and internal pressure measured during the extrusion process after addition of CNT to ABS (see Table 1). Even though the MFI of nanocomposites with CNT content higher than 4 wt % is extremely low, it has been possible to produce feedstock filaments by using twin screw extruder up to 8 wt % of CNT, reaching maximum values of internal pressure of about 46 bar and 120 Nm of torque.

### 3.2. Bulk Density

**Figure 3:**
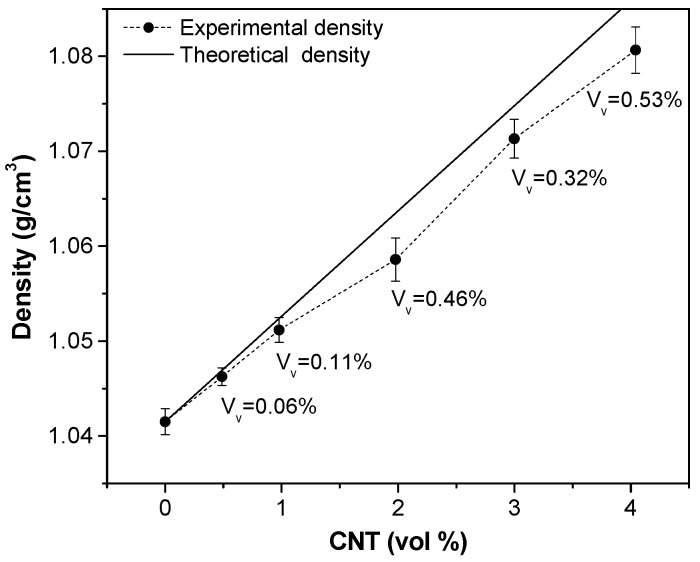
Experimental density values of ABS-CNT nanocomposite compared to theoretical density and voids fraction (*V*_v_).

Density of CNTs was estimated to be 2.151 ± 0.033 g/cm^3^ (see Supplementary Materials Figure S3). The bulk density of filaments is plotted in Figure 3 as a function of CNT volume fraction. The density of neat ABS filament is 1.042 g/cm^3^, which is consistent with the reported value in the materials technical data sheet [19]. Density of ABS/CNT composites increases almost linearly with rising fraction of CNT up to 1.081 g/cm^3^ at 8 wt % of CNT (corresponding to about 4 vol %). As it can be seen, the experimental density of ABS filled CNT nanocomposites is slightly lower than the theoretical density estimated by using the rule of mixture, which evidences the presence of microvoids, whose volume fraction (*V*_v_) is reported in Figure 3. Details of voids determination are reported in Supplementary Materials Section 2.

### 3.3. Morphological Analyses on Filaments and 3D-Printed Parts

The fracture surface of cryogenically broken filaments and 3D-printed specimens were analyzed by electron microscopy.

**Figure 4:**
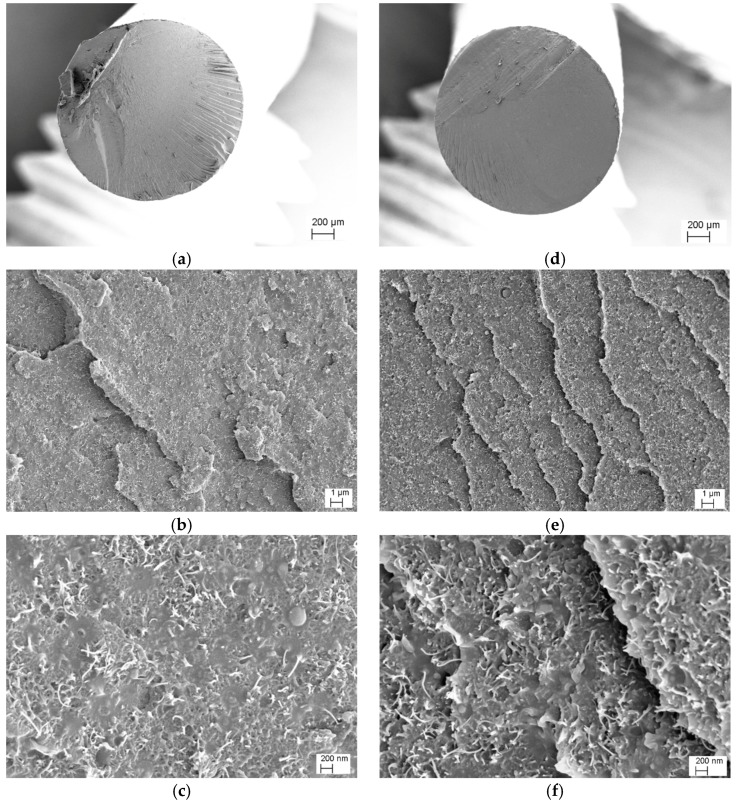
FESEM micrographs of CNT6 (**Left**) and CNT8 (**Right**) filaments at different magnifications (**a**,**d**) ×80, (**b**,**e**) ×10,000 and (**c**,**f**) ×50,000. Carbon nanotubes are identified in the form of small white lines in the highest magnification images.

Figure 4 illustrates the SEM images of ABS/CNT filaments with a CNTs content of 6 and 8 wt % at increasing magnification. Regarding the CNTs dispersion in both compositions, a homogenous distribution of single nanotubes in ABS matrix can be observed (no aggregates of nanotubes were detected). This means that the adopted two-steps process, consisting of mixing in an internal mixer followed by twin-screw extrusion, was capable to avoid the formation of nanofiller aggregates and to properly disperse CNTs in the ABS matrix. In addition, at high magnifications, a good adhesion level between CNT and ABS can be observed.

**Figure 5:**
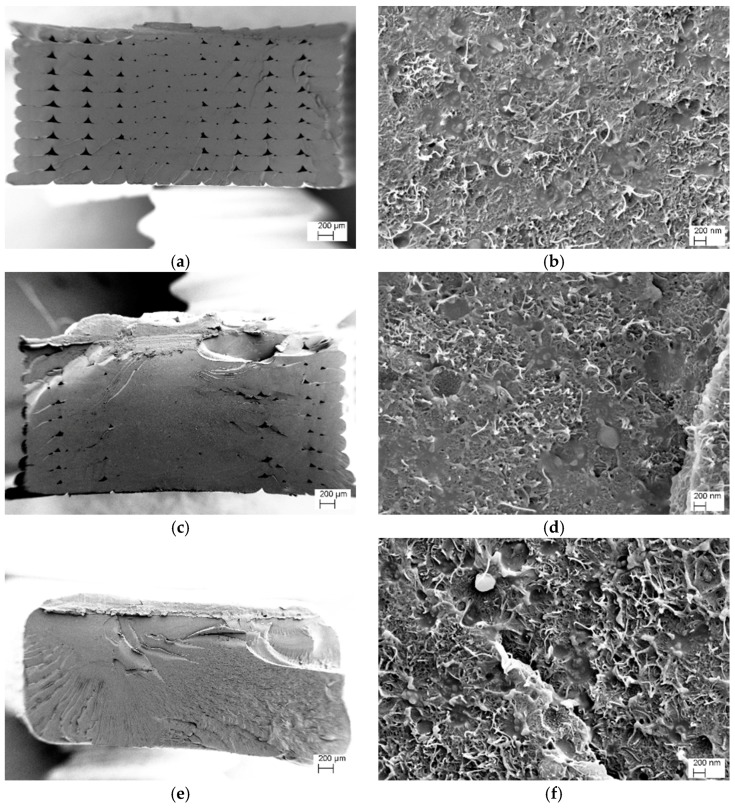
FESEM micrographs of 3D-printed dumbbell specimens printed from carbon nanotubes nanocomposites, CNT6-HC (**a**,**b**), CNT6-H45 (**c**,**d**) and CNT6-VC (**e**,**f**). Carbon nanotubes are identified in the form of small white lines in the high magnification images (see also Figure S7).

In Figure 5a–f, the cross-sections of FDM nanocomposite specimens at low and high magnifications are visualized. Moreover, for FDM specimens the presence of voids (about 3 and 1 vol % as observed from Figure 5a,c respectively) is documented. Also, uniform dispersion of nanofillers can be observed in Figure 5b,d,f for all FDM specimens at different build orientations. By using the ImageJ software, the diameter of nanotubes was estimated to be about 33 ± 3 nm for all specimens (average of ten measurements).

### 3.4. Thermal Degradation Behavior

**Figure 6:**
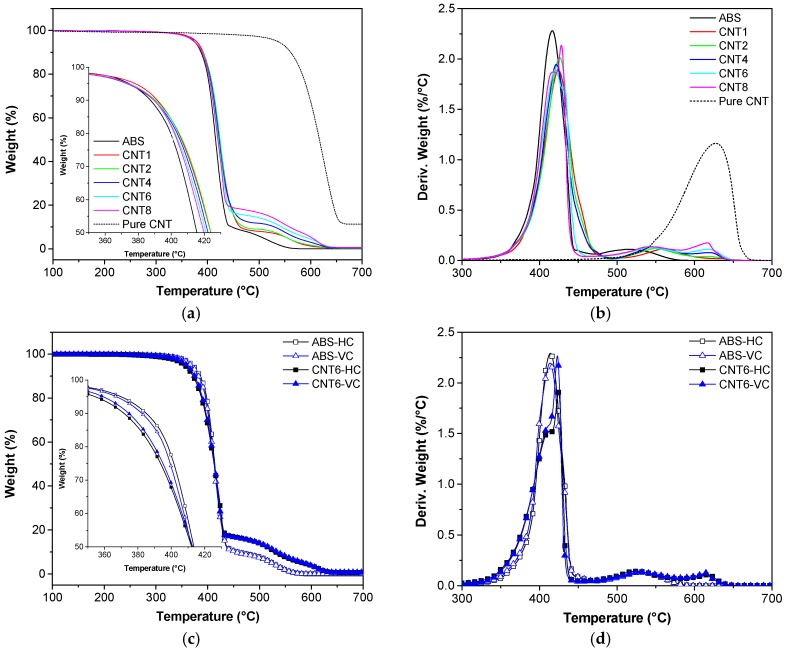
TGA curves of neat and nanofilled ABS filament and 3D-printed samples (HC and VC) under air atmosphere: (**a**,**c**) Residual mass as a function of temperature; (**b**,**d**) Derivative of the mass loss.

Thermal stability of ABS matrix and prepared composites was investigated by using thermal gravimetric analysis (TGA). Figure 6a,b depicts the TGA thermogram of neat ABS and CNT-filled composite filaments, while the most important parameters are summarized in Table 4. For the neat ABS in air environment two main degradation steps can be clearly observed at 416 °C and 514 °C, that could be attributed to the molecular chain scission and the oxidation of residual species, respectively [26,27]. On the other hand, neat CNTs showed one single decomposition step at around 627 °C. The onset temperature (*T*_onset_) and the maximum degradation temperature (*T*_d,max_) of the composites slightly increase with rising CNTs fraction up to a maximum value for 2 wt % of CNTs; afterwards they decrease. Similar behavior was also observed for other systems, such as polylactic acid/CNT, where it was attributed to possible aggregation and breakage of CNTs at elevated concentrations [28].

For CNT6 and CNT8 samples, it is possible to note that double peaks occurred between 420–430 °C. Moreover, an additional peak of nanocomposites with more than 4 wt % of CNT can be observed around 616–618 °C, which might be associated with the presence of CNT. The maximum mass loss rate (MMLR) in Figure 6b is progressively reduced by the presence of CNT since the nanofiller can hinder the diffusion of volatile products generated by polymer decomposition [27,28,29]. As reported in Table 4, the residue of tested composites at 700 °C increases with the CNT fraction. However, the residual mass is lower than the nominal amount of CNT because of the oxidation of CNT in the air in the course of experiments.

TGA thermograms reported in Figure 6c,d prove that 3D-printed specimens prepared at the different built orientations (HC and VC) exhibited a behavior similar to that observed for neat ABS filaments. However, as reported in Table 4, 3D-printed nanocomposite samples, i.e., CNT6-HC and CNT6-VC, showed a slightly lower *T*_onset_ than the corresponding neat ABS samples. The residue at 475 °C and 575 °C was considered to evaluate the CNTs content. In particular, the relative residue obtained after subtraction of ABS contribute fit quite well with the nominal CNT wt %.

### 3.5. Differential Scanning Calorimetry

**Figure 7:**
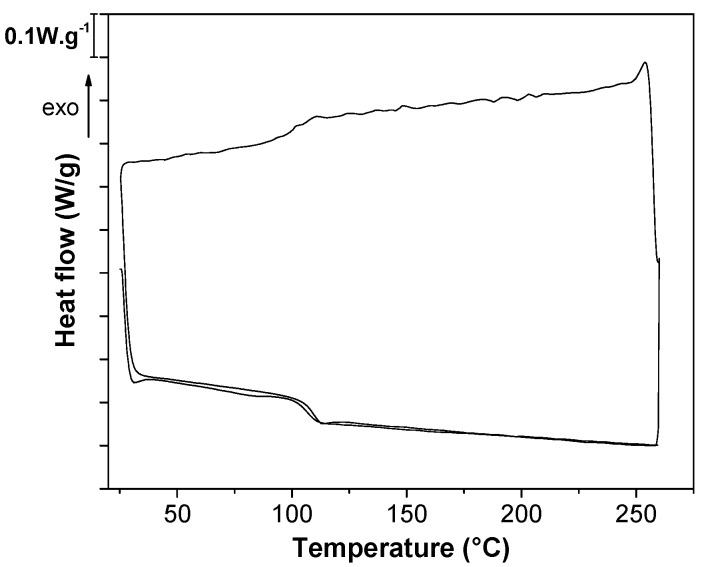
Representative DSC thermogram of CNT6 nanocomposite filament.

Representative DSC thermograms are shown in Figure 7 for CNT6 filament. All DSC thermograms of neat matrix ABS and of CTN filled composites are depicted in Figure S4 and were used for the determination of the glass transition temperature *T*_g_ (Table S2). The *T*_g_ values found for SAN phase in neat ABS and in CNT-filled ABS filaments are about 106 °C and 108 °C at the first and the second heating run, respectively, which means that the presence of CNT has no significant effects on *T*_g_ of ABS/CNT composites. The glass transition temperature of 3D-printed neat ABS (ABS-HC and ABS-VC) is slightly higher than that of neat ABS filament at the first heating run but similar in the second heating run. Moreover, the presence of nanotubes does not have significant effects on the glass transition temperature of nanocomposites in all the three steps of the cycle (first heating-cooling second heating). Also, Yang et al. reported only a slight increase in *T*_g_ promoted by single wall carbon nanotubes (SWCNT) dispersed in ABS [27].

### 3.6. Mechanical Behavior

#### 3.6.1. Quasi-Static Tensile Test

Tensile properties were measured for both filaments and fibers at various CNT contents. Table 5 shows an almost equivalent mechanical behavior of the different diameter extrudates (about 1.7 mm and 0.50–0.65 mm) with no direct dependence on the polymer orientation. Tensile energy to break (TEB) progressively decreases with CNT content and correspondingly the ductility factor for both filaments and fibers, especially above 4 wt % of nanofiller.

**Figure 8:**
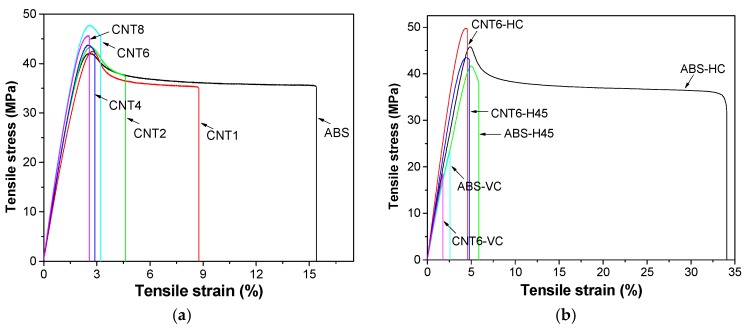
Tensile stress-strain curve of ABS and ABS-CNT filaments (**a**) and 3D-printed samples (**b**).

Representative stress-strain curves of filaments of neat ABS and its nanocomposites are reported in Figure 8. It is worth noting that CNT enhances both tensile modulus (*E*) and yield strength (σ_y_) of the composites (Table 5). At the highest concentration of nanotubes (8 wt %) the elastic modulus of ABS/CNT nanocomposites achieved a value 19% higher than that of ABS matrix. The highest σ_y_ was found for CNT6, while CNT8 shows a slight reduction in σ_y_ and almost brittle behavior. Therefore, ABS with 6 wt % of carbon nanotubes was an optimal compromise for FDM application.

Stress-strain curves of 3D-printed specimens are shown in Figure 8b and the resulting mechanical parameters are summarized in Table 6. Tensile modulus of H45 sample is comparable to that of HC sample probably because of good contact between bead extruded microfilaments and a lower fraction of voids in H45, as documented by SEM images (Figure 5a,c). Similarly enough, the lower yield strength of H45 with respect to that of HC is most probably due to internal orientations of deposited filaments as shown in Figure 1a,b. H45 or HC samples are expected to behave almost as isotropic or transversally isotropic materials. On the other hand, ABS-VC samples manifest a brittle behavior due to the weakness of interlayer bonding and the same behavior is even clearer for CNT6-VC samples because interlayer bonding could be significantly reduced by the higher viscosity in the molten state. Correspondingly, the ductility factor is zero, due to the absence of any toughening mechanism in the fracture process. In addition, the presence of CNTs resulted in an enhancement of both tensile modulus and yield stress for all FDM samples. The elastic modulus of ABS/CNT nanocomposites continuously increased up to 22%, 18% and 5% above that of unfilled ABS at the orientation of HC, H45 and VC, respectively. The highest yield stress can be observed in CNT6-HC sample owing to deposited filaments parallel to the applied load and the reinforcing effect of carbon nanotubes. As a side effect, the elongation at break of FDM composites samples was significantly reduced proportionally to the CNT content.

#### 3.6.2. Fracture Mechanism

Figure S5 presents the fractured surface of ABS and CNT6 3D-printed specimens broken in liquid nitrogen. For both HC and H45 samples, it is easy to observe along the thickness in *Z* direction 10 flattened parallel deposited bead microfilaments with a dimension of about 420 microns in width and 210 microns in height for ABS-HC (and about 410 micron and 210 micron for CNT6-HC). Taking into consideration the initial diameter of freely extruded fibers (Table 5), a further orientation of about 3.7 and 2.4 could be calculated during 3D-printing of the microfilament for ABS-HC and CNT6-HC, respectively (see Supplementary Materials Table S3).

At the same time, along with the sample width in the *Y* direction, HC evidenced 10 deposited microfilaments, whereas only 4 deposited parallel microfilaments could be observed in the external contours of H45 samples (2 on the right and 2 on the left). The inner microfilaments oriented at +45°/−45° could not be easily distinguished and an almost homogeneous zone appeared. For this reason, the similar stiffness of HC and H45 can be attributed to the combined effect of both the larger number of voids and orientation of microfilaments. On the other hand, for ABS-VC and CNT6-VC no traces of voids and of deposited microfilaments were observed in the cryo-fractured surface. These results have been attributed to the higher temperature of interlayer overlapping that is dependent on two factors: (i) the lower deposition rate (16 mm/s of VC sample with respect to 40 mm/s of other samples) and consequently the lower viscosity of deposited microfilament; and (ii) the lower time of deposition of the layer in VC samples with respect to HC samples (23 s vs. 46 s, respectively) and hence the higher temperature of the last deposited layer in VC sample (surface of deposition).

Difference is the case of a fractured cross-section of 3D-printed samples derived from the tensile test, as shown in Figure S6. The clear shape and size of the triangle between the deposited microfilaments (see Figure S6a) were observed due to the plastic deformation under tensile load. Moreover, some traces of microfilaments were partially evidenced in VC sample of both neat ABS and its nanocomposites, as shown in Figure S6c,f, which suggests a weak adhesion of the inter-layer bonding between microfilaments.

### 3.7. Creep Stability

**Figure 9:**
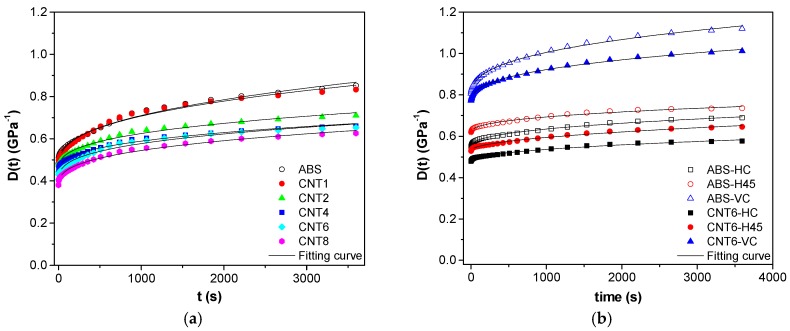
Creep compliance, D(t) at 30 °C, of neat ABS and nanocomposites as measured on (**a**) filaments at 3.9 MPa and (**b**) 3D-printed samples along different orientations at 3.0 MPa.

Figure 9a,b shows the creep compliance at 30 °C of neat ABS and composites found for (a) filaments and for (b) FDM samples. If no plastic deformation occurs, compliance of isothermal tensile creep, *D*_tot_(*t*), consists two components: elastic (instantaneous) *D*_el_ and viscoelastic (time-dependent) *D*_ve_, as defined in Equation (10).

*D*_tot_(*t*) = *D*_el_ + *D*_ve_(*t*) (10)

Incorporation of CNTs in ABS accounts for a pronounced reduction of both compliance components, as reported in Table 7. *D*_el_ is characterized by an almost linear decrease with CNTs fraction, which is in conformity with the inverse trend of tensile modulus (Table 5 and Table 6). For example, the composite with 8 wt % of the nanofiller showed *D*_el_ or *D*_tot,3600s_ by 21% or 26% lower than the neat matrix. For FDM samples, a similar effect of CNT on both elastic and viscoelastic creep compliance was observed: 6 wt % of the nanofiller in ABS matrix reduced the total compliance of nanocomposite by 16%, 12% and 10% for HC, H45 and VC respectively. 

The empirical Findley’s model (power law), summarized in Equation (11) was used to describe the viscoelastic creep response [30,31,32]:

*D*(*t*) = *D*_e_ + *kt^n^* (11)

where *D*_e_ is the elastic (instantaneous) creep compliance, *k* is a coefficient related to the magnitude of the underlying retardation process and *n* is an exponent related to the time dependence of the creep process. The fitting parameters for experimental creep data are summarized in Table 7. The fitting model was satisfactory, as *R*^2^ around 0.99 was found for all samples value. The addition of CNT reduced the creep compliance of composites; in particular, the value of parameter *D*_e_, for both filaments and 3D-parts are in good agreement with the values of *D*_el_. from Equation (10). The coefficient *n* reflects the kinetics of displacements of the segments of macromolecules in the viscous medium in the course of the creep and it was found to slightly decrease with the presence of CNT in ABS filaments.

### 3.8. Dynamic Mechanical Response and Coefficient of Thermal Expansion

**Figure 10:**
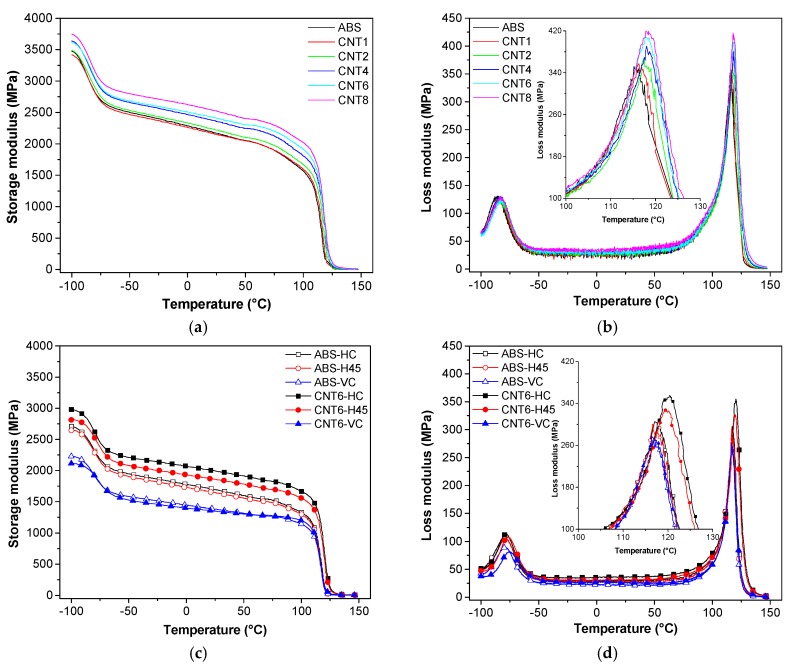
Dynamic mechanical thermograms of filament and 3D-printed samples (**a**,**c**) storage modulus (*E*’) and (**b**,**d**) loss modulus (*E*’’), of neat ABS and nanocomposite samples.

Figure 10 documents that ABS matrix and all composites show two transitions which can be identified with the glass transition of butadiene phase (B-phase; *T*_g1_ = −84 °C) and the glass transition of styrene–acrylonitrile phase (SAN phase; *T*_g2_ = 125 °C). Incorporation of CNT accounts for enhancement of the storage modulus of composites above that of ABS matrix, which becomes more pronounced at higher temperatures. For instance, at the highest concentration of CNT (8 wt %), the storage modulus of composite filament exceeds that of ABS by about 16% at 30 °C and by 897% at 130 °C.

Incorporated CNT also contributes to enhancing the dissipation of mechanical energy, as represented by the dynamic loss modulus. Moreover, the nanofiller also increases the glass transition temperatures of both butadiene and styrene–acrylonitrile phases by about 3 °C due to the hindering of segmental motions at the interface. Similar observations were also reported in prior papers [23,33].

As expected, the storage modulus of 3D-printed specimens at build parallel and ±45° orientations (HC and H45) is higher than that measured on samples with the VC orientation. The behavior observed for HC and H45 samples is related to the direction of the deposited filaments preferentially aligned and isotropic materials inclined at ±45° along the tensile applied load respectively, while the deposited layers in VC specimens are mostly oriented transversally to the tensile force. In general, the 3D-printed samples show storage modulus lower than original filaments due to the presence of voids and specific orientation of extruded microfilaments in 3D-printed samples (HC and VC).

The data summarized in Table 8, clearly show that the storage modulus of HC or H45 at 30 °C is enhanced by about 15% or 12% due to the addition of carbon nanotubes. The observed effect is even more pronounced at higher temperatures: at 130 °C the storage modulus of CNT6-HC or CNT6-H45 is 5 times higher than that of neat ABS-HC and ABS-H45. On the other hand, CNT do not exhibit any stiffening effect on storage modulus along VC orientation in the temperature range −50 to 30 °C, while a three-fold increase in the storage modulus can be observed at 130 °C.

The presence of carbon nanotubes also increases the glass temperature of CNT6-HC and CNT6-H45 by about 3 °C, which is identical with previously reported an increase in *T*_g_ for nanocomposite filaments (see Supplementary Materials Table S2).

The stiffness loss (*SL*_Tg_) at the glass transition temperature could be evaluated from the reduction of storage modulus before and after the transition (Δ*E*’), according to Equation (12) as a function of storage modulus at 30 °C.

*SL*_Tg_ = (Δ*E*’)/*E*’_30°C_ (12)

where Δ*E*’ represents the modulus variation from −100 °C to −50 °C, or from 90 °C to 130 °C, in the case of transition of butadiene or SAN phase, respectively (see data in Table 8).

In the zone of butadiene transition, the parameter *SL* was found to progressively decrease from about 0.45 (ABS matrix) up to 0.38 for CNT8, in dependence on the content of CNT for all nanocomposite samples (both filaments and 3D-printed parts) as indication of the role of nanofiller in the relative stiffening at room temperature. On the other hand, the stiffness loss at the main glass transition (*T*_g_ of SAN phase) is almost linearly increasing with CNT content, from 0.80 (ABS filament) to about 0.84 for CNT8 filaments and it depends on the stiffening of rubbery phase above *T*_g_.

**Figure 11:**
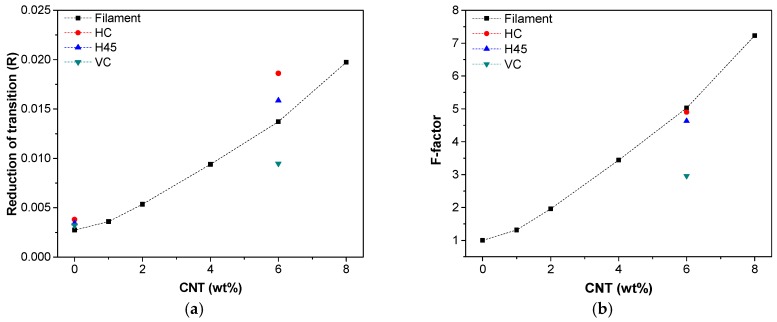
Reduction of main transition of storage modulus-*R* (**a**) and *F*-factor (**b**) as function of CNT nanofiller loading measured on filaments and 3D-printed samples (HC, H45 and VC).

Figure 11a,b shows the reduction of the main transition of storage modulus (*R*) and *F*-factor [23,24] which are plotted as functions of the CTN fraction. In Figure 11a, the stiffening effect of CNTs in the rubbery phase above *T*_g_ of SAN is well documented. In particular, this effect seems to be more pronounced for FDM samples (HC and H45) with respect to filaments CNT6, probably owing to the higher orientation and adhesion/dispersion of carbon nanotubes in FDM process.

Moreover, the *F*-factor represents a relative measure of modulus in the temperature interval of the glass transition, assuming that modulus at glassy state is dominated by the strength of intermolecular forces when polymer chains and nanofillers are packed [23]. Thus, the higher *F*-factor, the higher the effectiveness of the filler. Figure 11b presents the increase in the *F*-factor of filaments with the fraction of CNT and it confirms the relative effectiveness of CNT nanofiller with its fraction in composites in the rubbery phase.

For FDM sample (HC and H45), the reinforcing efficiency is slightly lower than filament at 6 wt % of CNT, maintaining almost the same adhesion level of nanofiller and matrix during FDM process. On the other hand, VC sample shows a different effect since the properties of these specimens are mainly dependent on the inter-layer matrix adhesion and mostly independent on the compatibility of the polymer chains and nanofiller.

**Figure 12:**
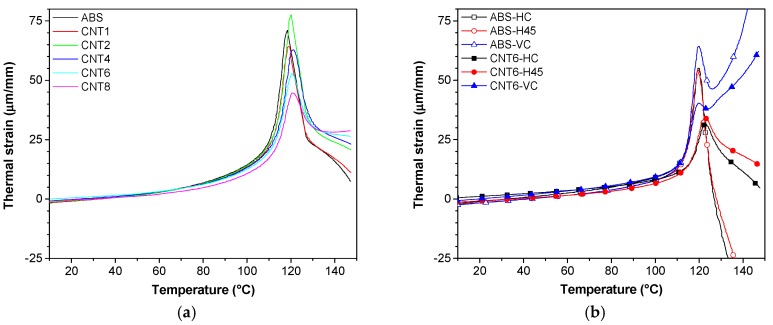
Thermal strain of neat ABS and nanocomposite samples as measured on filaments (**a**) and 3D-printed samples (**b**) along different orientations (HC, H45 and VC).

Thermal strain of ABS/CNT filaments is plotted in Figure 12a and the coefficient of thermal expansion of all samples is reported in Table 9. The thermal strain of composite filaments exhibited the linear trend up to 100 °C, i.e., approximately to the glass transition temperature. The steep increment of thermal strain indicates the transition from the glassy state to the rubbery state with the much higher mobility of polymer chains. Above 120 °C, the thermal strain showed negative slope with respect to the temperature scale due to some shrinkage of the polymer chains orientated during extrusion. Incorporated CNT markedly reduced the coefficient of thermal expansion (see Table 9). As expected, the composite with the highest concentration of CNT shows the largest drop of the coefficient of thermal expansion, i.e., 79.6 for ABS to 52.3 × 10^−6^/K for CNT8 at the room temperature (20~50 °C) and from −891 for ABS to −51 × 10^−6^/K for CNT8 at the temperature (130~150 °C).

In the temperature interval 20/50 °C, FDM specimens (HC, H45 and VC) printed from neat ABS, exhibit CLTE values of 85.8, 74.5 and 79.1 × 10^−6^/K, respectively (see Table 9). The presence of CNTs accounts for a reduction of the CLTE of FDM specimens by 31% or 27% for HC or H45 but no effect was observed for VC build orientation.

### 3.9. Electrical Behavior

#### 3.9.1. Electrical Resistivity

**Figure 13:**
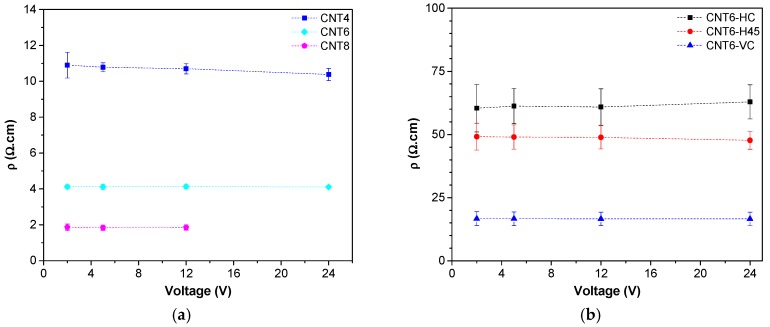
Electrical resistivity of ABS nanocomposites: (**a**) filament and (**b**) 6 wt % filled nanocomposites with different 3D printing as a function of the applied voltage.

The previous text has illustrated how CNT affects mechanical properties of prepared composites but most important effects of CNT can be expected in the field of electrical properties. Improvements of conductivity and electrical properties by incorporated CNT in different polymer, such as polyamide [34], polypropylene [35], polylactide [28] and ABS [18,29,36] have been documented in literature According to the technical data sheet [19], the volume resistivity of neat ABS bulk materials is 10^15^ Ω·cm. Our measurements reveal that the volume resistivity of the composites significantly decreases with at least 4 wt % of nanofiller (see Figure 13), whereas at CNTs fractions up to 2 wt %, the materials still exhibit an insulating behavior and filaments could not be tested by means of the four probes configuration.

The incorporation of CNTs decreases the electrical resistivity of filaments to about 11 Ω·cm, 4.1 Ω·cm and 1.8 Ω·cm for CNT4, CNT6 and CNT8, respectively. The volume resistivity of all samples is directly dependent on the CNTs content and independent on the applied voltages (Figure 13a), which suggests that these nanocomposites behave as ohmic conductors. It is also worth noting that CNT8 filaments could not be tested at 24 V due to the high resistive heating effect. 

The resistivity of 3D-printed samples CNT6-H45 and CNT6-VC shown in (Figure 13b) is independent of applied voltages and is higher than the correspondent filament. This partial reduction of conductivity not only in comparison with single filaments but also with compression molded specimens at the same composition (see Supplementary Materials Figure S9) could be attributed to the internal features of FDM samples. Moreover, it should be noted that CNT6-HC shows the highest resistivity, whereas CNT-VC the lowest. These results could be related to the better contact between deposited bead microfilaments, resulting in higher conductivity of samples; these findings are in good conformity with the documentation of SEM images (Figure 5e), where VC-CNT6 specimens exhibit better and extensive contacts between the layer of deposited microfilaments in the direction of electrical measurements.

Similarly, in literature, Zhang et al. reported that the resistivity of 3D-printed components was found lower than the pristine 3D-printing fibers and the results were also confirmed to be highly dependent on the contact resistivity by numerical simulation method [10].

In order to understand the electrical behavior of composite filaments and to evaluate the effect of CNT orientation in ABS, the most conductive filaments (i.e., CNT6 and CNT8) were compression-molded for the production of homogeneous plates (resistivity results are shown in Figure S9). It worth noting that the electrical resistivity of CNT8-0, CNT8-45 and CNT8-90 was found to directly depend on the angles of filament orientation in the plate (Figure S8c). The resistivity of CNT8-0 is similar to that of CNT8 filament owing to the almost identical filaments orientation, whereas CNT8-90 leads to the lower level of filaments alignment with respect to the electrical field. The higher the angle, the higher the resistivity. And the same for specimens CNT6-0, CNT6-45 and CNT6-90, at resistivity even higher. From these findings, the electrical resistivity of filaments could be considered a quasi-isotropic behavior of materials with partial random oriented CNT.

However, after FDM process the conductivity of 3D-printed fibers (see Table S4) slightly increases, so that is comparable to that of plate samples. The results suggest that the orientation CNT during the extrusion contributes to the reduction of the resistivity of the composites. A similar effect was also observed for the composites with graphene oxide [8] and carbon black [10].

**Figure 14:**
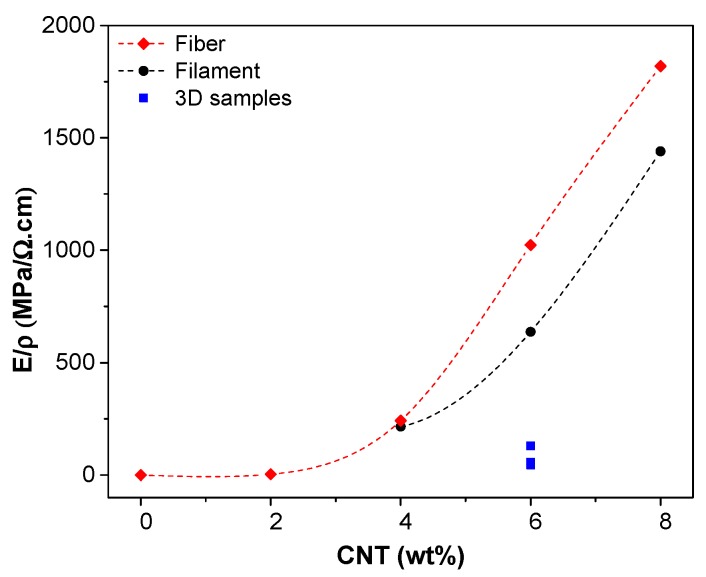
The ratio modulus/resistivity reference at 5 V as a function of CNT % for filament (●), fiber (♦) and 3D samples (■).

The beneficial effect of CNTs could be summarized in the double results to increase the stiffness and to reduce the electrical resistivity of ABS nanocomposites, considering the experimental ratio modulus/resistivity, expressed as MPa/Ω·cm. Figure 14 depicts this double effect, revealing that filaments and fibers exhibited the best behavior especially with a CNT of 6–8 wt %. The relative lower values of 3D-printed specimens directly depend on the specific FDM process.

#### 3.9.2. Surface Temperature under Applied Voltage

**Figure 15:**
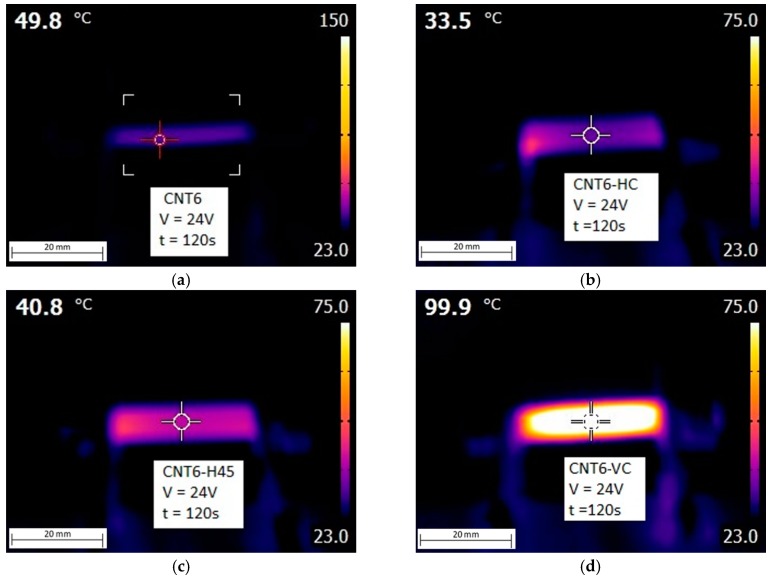
Results of thermal imaging upon voltage application at 24 V at 120 s: CNT6 filament (**a**); CNT6-HC (**b**); CNT6-H45 (**c**) and CNT6-VC (**d**).

**Figure 16:**
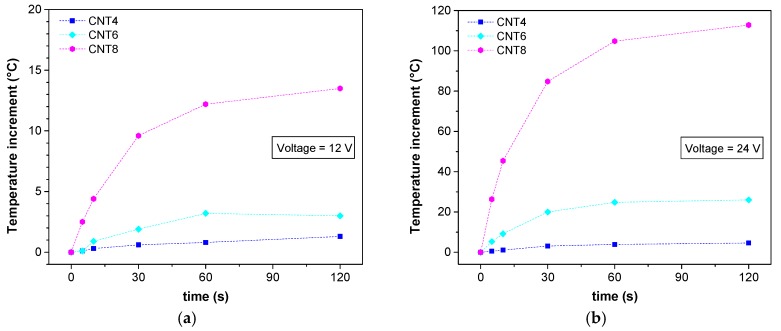

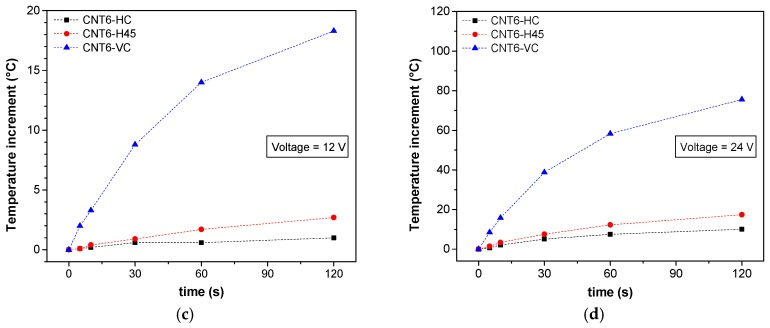
Increment of surface temperature upon a voltage of 12 V (**a**,**c**) and 24 V (**b**,**d**) for ABS nanocomposites filaments and 3D-printed samples with different CNT loading at room temperature of 23 °C.

The measurement of Joule’s heating upon voltage application of the samples with different fractions of CNTs was performed for 12 and 24 V which are commonly reached by batteries for automotive applications. In Figure 15, we can see the representative images of the evolution of surface temperature upon voltage application to CNT6 filament and FDM samples. The highest temperature was obviously concentrated in the center of samples due to the cooling effect at the border of samples due to the relatively low thermal conductivity. We monitored the evolution of the surface temperature as a function of the voltages, the time and the composition of nanocomposite materials. As shown in Figure 16a,b the increment of the temperature of all samples under both voltages (12 V and 24 V) seems to reach the plateau after 60 s. Obviously, the higher the applied voltage, the higher the increase in temperature. Besides, the resistivity of composite materials of ABS/CNT filaments evidences a good correlation with the increase of the temperature. The higher the conductivity, the higher the increase in the surface temperature of samples due to the dissipation of thermal energy. For example, CNT4 sample does not show any significant increase in temperature, whereas a rather high increase in temperature can be seen for CNT8. It is worth noting that, at applied voltage of 24 V for 120 s (Figure 16b), the generated surface temperature of CNT8 sample exceeds the glass transition temperature of ABS. Therefore, in order to avoid thermal degradation of materials during prolonged voltage application, between the various ABS materials studied in this research, the CNT6 samples appeared the most convenient nanocomposite materials for electro-conductive applications.

The electrical measurements of 3D-printed samples with CNT contents of 6 wt % built with different orientations were performed by using the same two voltages applied to the filaments (12 V and 24 V). Surface temperature under applied voltage shows good correlation with resistivity measurements. Lower resistivity resulted in a higher increment of temperature, e.g., CNT6-VC reached the highest temperature of about 100 °C after 120 s. However, the local temperature of all FDM samples achieved via the Joule’s effect remains below the glass transition temperature of ABS, which allows us to presume good thermal stability of produced nanocomposite materials in electrical applications.

## 4. Conclusions

Carbon nanotubes (in fractions up to 8 wt %) were directly melt compounded with relatively high viscosity ABS matrix by using a completely solvent-free process. Subsequently, by using a twin-screw extruder, composite filaments were appositely extruded for application in 3D printing with fused deposition modelling. 

The optimum CNT fraction for fused deposition modelling process was found to be 6 wt %. Thermal, mechanical and electrical properties of neat ABS and ABS/CNT composites have been investigated on produced filaments and 3D-printed parts. CNT has the positive effect on the resistance to long-lasting loads due to the reduction of creep compliance. Besides, the enhancement of both tensile modulus and strength was found for filaments and FDM products, except for vertical 3D built specimens. On the other hand, elongation at break of the composites was reduced in proportion to the CNT fraction. The presence of CNT also promoted the thermal stability of 3D-printed parts due to the reduction in coefficient of thermal expansion.

Electrical conductivity of 3D-printed samples was markedly incremented but a partial loss in conductivity with respect to filament nanocomposite was also observed. Moreover, the resistivity of 3D-printed parts is highly dependent on the build microfilaments orientation, which consequently leads to different surface temperature increment under applied voltages. For FDM-printed parts, the carbon nanotubes in playing the best reinforcement in thermal mechanical behavior for HC and H45 orientation but less effective in electrical properties.

## Figures and Tables

**Table 1:** Processing parameters of twin screw extruder for the production of ABS and ABS/CNT nanocomposite filaments.

Samples	Pressure (bar)	Torque (Nm)	Screw Speed (rpm)	Collection Rate (m/min)	Output (g/h)
ABS	16.9	40.4	5	1.00	137.6
CNT1	17.1	38.4	5	1.00	134.6
CNT2	21.7	45.9	5	1.00	137.7
CNT4	28.0	66.8	5	1.00	138.6
CNT6	44.2	100.1	5	1.15	139.6
CNT8	45.7	119.6	4.5	0.88	122.1

**Table 2:** 3D-printing parameters for each build orientation.

Samples	Infill Type	Raster Angle (°)	Printing Speed (%)
HC	Concentric	[0, 0]	100
H45	Rectangular	[+45, −45]	100
VC	Concentric	[0, 0]	40

**Table 3:** Bulk density and linear density of ABS and ABS/CNT nanocomposite during filament extrusion and 3D fiber production. Extrusion and 3D printing draw ratio.

Samples	CNT Content (wt %)	Bulk Density (g/cm^3^)	Filament Linear Density (tex)	Filament Extrusion *OF*_E_^1^	Fiber Linear Density (tex)	3D-Printing *OF*_3D_^2^	Fiber Swelling *DS* ^3^	Fiber *OF*_T_^4^
ABS	0	1.042 ± 0.001	2389 + 139	1.09	349 ± 17	7.1	2.6	7.7
CNT1	1	1.046 ± 0.001	2256 + 18	1.15	290 ± 7	8.1	2.2	9.3
CNT2	2	1.051 ± 0.001	2287 + 71	1.14	267 ± 5	8.8	2.0	10.1
CNT4	4	1.059 ± 0.002	2534 + 83	1.04	231 ± 3	11.2	1.7	11.6
CNT6	6	1.071 ± 0.002	2425 + 64	1.11	224 ± 3	11.1	1.7	12.2
CNT8	8	1.081 ± 0.002	2387 + 64	1.12	219 ± 2	11.3	1.6	12.7

^1^ Draw ratio of filament (extrusion) see Equation (6). ^2^ Draw ratio in 3D printing see Equation (8). ^3^ Fiber swelling. ^4^ Total Draw ratio of fiber (extrusion and 3D printing) see Equation (9).

**Table 4:** TGA data of pure ABS and its nanocomposites in an air atmosphere.

Samples	*T*_onset_ (°C)	*T*_max1_ (°C)	*T*_max2_ (°C)	*T*_CNT_ (°C)	Residue at (wt %)			Relative Residue at (wt %) ^1^	
					475 °C	575 °C	700 °C	475 °C	575 °C
ABS	394.0	416.5	514.4	/	7.9	0.1	0.0	0.0	0.0
CNT1	397.5	424.6	560.4	/	8.8	2.8	0.2	0.9	2.7
CNT2	399.4	426.2	548.6	/	9.7	3.0	0.2	1.8	2.9
CNT4	396.9	421.5	547.1	618.2	12.6	4.8	0.4	4.7	4.7
CNT6	394.8	420.6	542.2	617.5	15.4	6.6	0.6	7.5	6.5
CNT8	394.6	428.3	544.1	616.6	17.8	8.2	0.7	9.9	8.1
Pure CNT	576.3	/	/	627.3	97.7	84.6	11.3	/	/
ABS-HC	391.9	414.0	531.8	/	9.2	0.2	0.0	0.0	0.0
ABS-VC	391.0	415.2	535.6	/	8.9	0.2	0.0	0.0	0.0
CNT6-HC	382.3	424.5	525.3	618.0	15.8	5.4	0.6	6.6	5.2
CNT6-VC	388.0	423.1	529.0	613.6	15.6	5.7	0.8	6.7	5.5

^1^ calculated according to Equation (1).

**Table 5:** Quasi-static tensile properties of ABS and its nanocomposite of filaments and single fiber (f) produced by twin screw and FDM extrusion, respectively.

Samples	Filament Diameter (mm)	*E* (MPa)	σ_y_ (MPa)	σ_b_ (MPa)	ε_b_ (%)	TEB ^1^ (MJ/m^3^)	Ductility Factor ^2^ P/TEB
ABS	1.725 ± 0.049	2207 ± 65	42.8 ± 1.9	35.0 ± 0.4	25.6 ± 15.8	8.94 ± 5.61	0.907 ± 0.040
CNT1	1.679 ± 0.007	2132 ± 63	42.9 ± 0.4	35.1 ± 0.3	7.9 ± 2.4	2.59 ± 0.86	0.713 ± 0.104
CNT2	1.684 ± 0.025	2226 ± 48	43.3 ± 0.3	37.8 ± 1.8	4.4 ± 1.2	1.36 ± 0.45	0.464 ± 0.153
CNT4	1.765 ± 0.026	2320 ± 74	43.4 ± 0.9	41.9 ± 1.7	2.6 ± 0.3	0.65 ± 0.16	0.099 ± 0.083
CNT6	1.712 ± 0.035	2625 ± 55	47.1 ± 0.5	44.6 ± 1.0	3.2 ± 0.5	1.04 ± 0.24	0.273 ± 0.158
CNT8	1.702 ± 0.016	2650 ± 125	46.8 ± 1.2	46.5 ± 1.1	2.5 ± 0.2	0.73 ± 0.10	0.046 ± 0.065
f-ABS	0.648 ± 0.021	1918 ± 105	40.4 ± 0.9	33.6 ± 0.8	52.8 ± 27.2	18.5 ± 9.60	0.944 ± 0.023
f-CNT1	0.591 ± 0.012	1801 ± 122	39.3 ± 1.7	35.8 ± 1.5	6.4 ± 3.2	2.00 ± 1.20	0.463 ± 0.259
f-CNT2	0.567 ± 0.007	2033 ± 142	40.4 ± 0.5	33.6 ± 0.8	4.9 ± 1.2	1.53 ± 0.44	0.371 ± 0.168
f-CNT4	0.528 ± 0.001	2035 ± 58	42.9 ± 1.4	40.8 ± 2.4	4.9 ± 1.2	1.59 ± 0.48	0.335 ± 0.220
f-CNT6	0.515 ± 0.003	2099 ± 124	44.9 ± 1.3	44.1 ± 1.6	4.1 ± 0.6	1.29 ± 0.23	0.124 ± 0.098
f-CNT8	0.506 ± 0.005	2147 ± 80	47.1 ± 0.6	46.9 ± 0.9	4.0 ± 0.7	1.31 ± 0.31	0.096 ± 0.075

^1^ Total energy to break. ^2^ Ratio between the propagation energy (P) from the yield to break point, with respect to TEB.

**Table 6:** Quasi-static tensile properties of ABS and its nanocomposite of FDM samples.

Samples	*E* (MPa)	σ_y_ (MPa)	σ_b_ (MPa)	ε_b_ (%)	TEB ^1^ (MJ/m^3^)	Ductility Factor ^2^ *P*/TEB
ABS-HC	2235 ± 170	45.7 ± 0.5	31.9 ± 1.7	30.0 ± 10.4	10.7 ± 3.76	0.866 ± 0.077
ABS-H45	2308 ± 112	41.1 ± 0.9	37.9 ± 1.6	5.3 ± 0.5	1.30 ± 0.16	0.204 ± 0.120
ABS-VC	2077 ± 44	/	22.0 ± 4.4	2.4 ± 0.7	0.30 ± 0.10	0
CNT6-HC	2735 ± 158	49.6 ± 0.6	49.2 ± 0.6	4.5 ± 0.2	1.35 ± 0.10	0.048 ± 0.044
CNT6-H45	2739 ± 268	43.2 ± 0.3	42.6 ± 0.4	4.6 ± 0.3	1.19 ± 0.11	0.054 ± 0.056
CNT6-VC	2181 ± 51	/	18.7 ± 1.5	1.9 ± 0.1	0.18 ± 0.03	0

^1^ Total energy to break. ^2^ Ratio between the propagation energy (*P*) from the yield to break point, with respect to TEB.

**Table 7:** Elastic (*D*_el_), viscoelastic *D*_ve_ (*t* = 3600 s) and total *D* (*t* = 3600 s) creep compliance at 3600 s and fitting parameters (Equation (11)) of ABS and its nanocomposites as measured on filaments and FDM samples.

Samples	*D*_el_ (GPa^−1^)	*D*_ve,3600s_ (GPa^−1^)	*D*_tot,3600s_ (GPa^−1^)	*D*_e_ (GPa^−1^)	*K* (GPa^−1^ s^−n^)	*n*	*R*^2^
ABS	0.482	0.369	0.851	0.488	0.012	0.419	0.9924
CNT1	0.471	0.362	0.833	0.460	0.020	0.364	0.9889
CNT2	0.450	0.261	0.710	0.447	0.016	0.345	0.9912
CNT4	0.436	0.225	0.660	0.436	0.014	0.342	0.9920
CNT6	0.405	0.246	0.652	0.393	0.020	0.319	0.9844
CNT8	0.380	0.246	0.626	0.374	0.016	0.347	0.9925
ABS-HC	0.521	0.169	0.689	0.547	0.005	0.402	0.9951
ABS-H45	0.587	0.148	0.735	0.616	0.005	0.392	0.9910
ABS-VC	0.756	0.364	1.120	0.783	0.019	0.355	0.9980
CNT6-HC	0.454	0.123	0.577	0.479	0.002	0.469	0.9905
CNT6-H45	0.501	0.145	0.645	0.531	0.002	0.503	0.9917
CNT6-VC	0.729	0.283	1.012	0.758	0.013	0.366	0.9981

**Table 8:** Dynamic mechanical properties of neat ABS and its nanocomposites as measured on filaments and FDM samples.

Samples	Storage Modulus					Damping Peaks		Loss Modulus of SAN Peak		Stiffness Loss ^1^ at *T*_g_	
	−100 °C (MPa)	−50 °C (MPa)	30 °C (MPa)	90 °C (MPa)	130 °C (MPa)	B-Phase *T*_g1_	SAN-Phase *T*_g2_	*E*”_peak_ (MPa)	*T*_peak_	*SL T*_g1_	*SL T*_g2_
ABS	3474	2503	2145	1711	4.7	−84.9	122.7	347	115.6	0.453	0.795
CNT1	3417	2468	2129	1729	6.2	−83.6	123.2	355	116.3	0.446	0.809
CNT2	3487	2531	2197	1809	9.7	−82.4	125.5	356	117.6	0.435	0.819
CNT4	3636	2662	2342	1952	18.3	−82.9	126.0	380	118.4	0.416	0.826
CNT6	3614	2685	2390	2044	28.0	−81.5	124.6	406	118.0	0.389	0.844
CNT8	3747	2799	2496	2139	42.2	−82.2	125.6	419	118.4	0.380	0.840
ABS-HC	2709	1948	1678	1415	5.4	−78.6	124.9	304	117.2	0.454	0.840
ABS-H45	2646	1904	1631	1384	4.7	−78.0	124.8	293	116.9	0.455	0.846
ABS-VC	2229	1583	1367	1207	3.9	−77.9	124.1	267	117.0	0.473	0.880
CNT6-HC	2980	2211	1977	1749	32.6	−77.5	127.6	354	120.3	0.389	0.868
CNT6-H45	2813	2080	1845	1632	25.9	−75.3	127.0	326	120.0	0.397	0.871
CNT6-VC	2114	1527	1338	1245	11.8	−73.9	123.4	270	117.1	0.439	0.922

^1^ stiffness loss (*SL*) calculated according to Equation (12).

**Table 9:** Coefficients of linear thermal expansion (CLTE) and linear thermal deformation (CLTD) of ABS and its nanocomposites as measured on filament and FDM samples (see Equation (4) for detail).

Sample	CLTE (×10^−6^/K)				CLTD (×10^−6^/K)
	Δ*T*_1_ = −50/−20 °C	Δ*T*_2_ = 20/50 °C	Δ*T*_3_ = 70/90 °C	Δ*T*_4_ = 108/113 °C	Δ*T*_5_ = 130/150 °C
ABS	49.7 ± 0.2	79.6 ± 0.4	262.9 ± 2.1	2350 ± 68	−891 ± 11
CNT1	51.6 ± 0.3	78.5 ± 0.5	246.1 ± 1.8	1880 ± 55	−678 ± 55
CNT2	48.4 ± 0.1	70.3 ± 0.4	250.8 ± 2.0	1770 ± 54	−491 ± 7
CNT4	43.2 ± 0.1	67.5 ± 0.5	223.8 ± 2.1	1450 ± 44	−465 ± 7
CNT6	38.1 ± 0.1	55.9 ± 0.3	207.0 ± 2.1	1290 ± 26	−206 ± 7
CNT8	33.7 ± 0.1	52.3 ± 0.2	191.3 ± 1.9	1150 ± 21	−51 ± 7
ABS-HC	61.0 ± 0.1	85.8 ± 0.3	156.6 ± 1.2	1040 ± 41	−4860 ± 50
ABS-H45	58.0 ± 0.2	74.5 ± 0.2	146.5 ± 1.1	1210 ± 36	−3620 ± 32
ABS-VC	61.0 ± 0.2	79.1 ± 0.2	147.3 ± 0.9	1330 ± 36	3310 ± 67
CNT6-HC	40.2 ± 0.1	59.0 ± 0.2	106.7 ± 0.9	479 ± 11	−805 ± 4
CNT6-H45	41.1 ± 0.1	54.0 ± 0.2	114.3 ± 1.0	587 ± 16	−506 ± 2
CNT6-VC	57.8 ± 0.1	79.4 ± 0.3	139.7 ± 0.8	1010 ± 28	1090 ± 12

## Supplementary Materials

The following are available online at www.mdpi.com/2079-4991/8/1/49/s1, Figure S1: Schematic of 3D-printed Parallelepiped: (a) horizontal concentric (HC); (b) horizontal 45° angle (H45) and (c) vertical concentric (VC), Figure S2: 3D-printed dumbbells, resistivity and resistivity heating specimens of ABS and ABS-CNT nanocomposites, Figure S3: Density of carbon nanotube measured through a Micromeritics^®^Accupyc 1330 helium pycnometry (23 °C) with 10 cm^3^ chamber, Figure S4: DSC thermogram of neat ABS and its ABS/CNT nanocomposites: (a) filaments; (b) 3D-printed samples, Figure S5: Frozen fracture of cross-section of 3D-printed dumbbells: (a) ABS-HC; (b) ABS-H45; (c) ABS-VC; (d) CNT6-HC; (e) CNT6-H45 and (f) CNT6-VC, Figure S6: Tensile fracture of cross-section of 3D-printed dumbbells: (a) ABS-HC; (b) ABS-H45; (c) ABS-VC; (d) CNT6-HC; (e) CNT6-H45 and (f) CNT6-VC, Figure S7: SEM micrographs of 3D-printed dumbbell specimens of CNT6-HC with indicating CNTs (red arrow). Figure S8: Summary of preparation of filament plate with the mold 50 mm × 50 mm × 1.0 mm starting with filaments at 6 and 8 wt % of CNT: (a) before compression and (b) after compression; (c) Schematic of samples at the different angles (0, 45 and 90°) for measuring electrical resistivity (see Figure S8), Figure S9: Electrical volume resistivity of ABS 6 wt % and 8 wt % filled nanocomposites of filament plates at different angles (0, 45 and 90°) as a function of the applied voltage, Table S1: Dimensions and processing parameters of FDM specimens, Table S2: Glass transition temperatures (*T*_g_) of styrene–acrylonitrile phase in ABS and in nanocomposite (from inflection point of DSC thermogram). Table S3: Evaluation of orientation factor in microfilament during 3D printing (according Equation (S4)). Table S4: Volume resistivity of different kinds of ABS-CNT samples at an applied voltage of 5 V.
