# Supplementary material for: Filaments Production and Fused Deposition Modelling of ABS/Carbon Nanotubes Composites
Source: Nanomaterials (Basel). 2018 Jan 18;8(1):49. doi: 10.3390/nano8010049 (PMC5791136; doi:10.3390/nano8010049)
Supplement: Supplementary file 1 [file nanomaterials-08-00049-s001.pdf]

Filaments Production and Fused Deposition Modelling of ABS/Carbon Nanotubes Composites

Sithiprumnea Dul, Luca Fambri and Alessandro Pegoretti *

1. FDM Preparation of 3D-printed Specimens

Table S1. Dimensions and processing parameters of FDM specimens.

Sample	X (mm)	Y (mm)	Z (mm)	D deposition time of single layer (sec)	Number of layers	total time [2] (min)	Analysis
**Dumbbell**							
HC	75	4-12.5 [1]	2	43	10	8.0	Tensile test
H45	75	4-12.5 [1]	2	33	10	6.0	Tensile test
VC	4-12.5 [1]	2	75	23	375	183.0	Tensile test
**Parallelepiped**							
	25	4	1	10	5	1.2	Creep
	25	4	2	10	10	2.0	DMA
HC	25	6	2	12	10	4.0	Resistivity
	50	6	2	23	10	7.0	Resistive heating
	25	4	1	10	5	1.2	Creep
	25	4	2	10	10	2.0	DMA
H45	25	6	2	11	10	4.0	Resistivity
	50	6	2	16	10	5.0	Resistive heating
	1	4	25	10	125	35.0	Creep
	2	4	25	22	125	77.5	DMA
VC	2	6	25	11	125	37.5	Resistivity
	2	6	50	11	250	75.0	Resistive heating

[1] min and max values of specimen's width are reported. [2] For the production of one FDM specimen taking into account non-print moves of the nozzle.

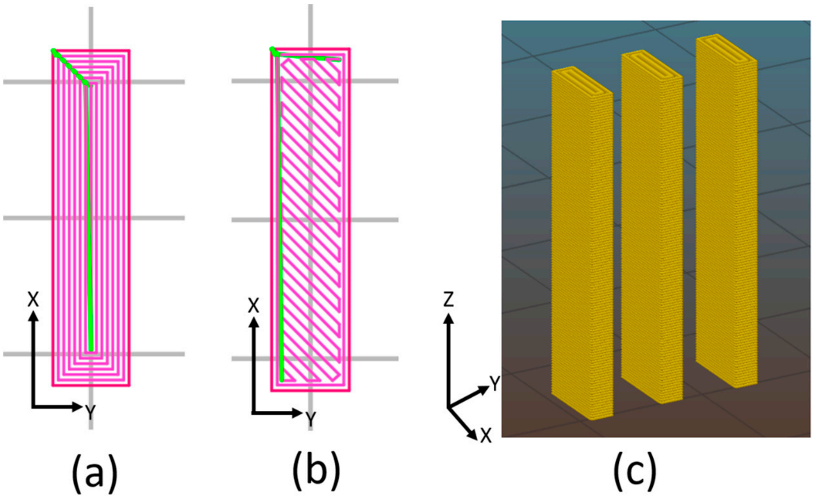

Figure S1. Schematic of 3D-printed Parallelepiped: (**a**) horizontal concentric (HC), (**b**) horizontal 45°angle (H45) and (**c**) vertical concentric (VC).

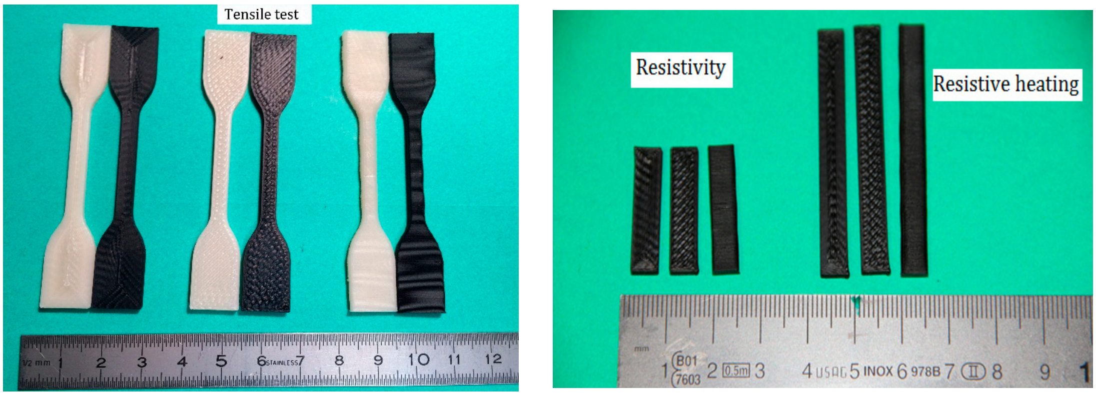

Figure S2. 3D-printed dumbbells, resistivity and resistivity heating specimens of ABS and ABS-CNT nanocomposites.

2. Density Measurement and DSC Analysis

Density measurements of bulk composites filaments were performed by using solvent ethanol-water (a concentration of 96 wt % and density of 0.802 g/cm^3) at room temperature, and at least three replicated specimens for each sample, in conformity of ASTM D782-13. The measurement was adopted through Equation S1.

$$\rho_{\exp} = \frac{m_{air} \times \rho_{ethanol}}{m_{air} - m_{ethanol}} \tag{S1}$$

where m_{air} and $m_{ethanol}$ are the mass of samples in air and ethanol respectively.

In order to compare the experimental results, theoretical density of composites was predicted based on the rule of mixture following to Equation S2.

$$\rho_{th} = \rho_m \times V_m + \rho_f \times V_f \tag{S2}$$

where ρ_c, ρ_m, ρ_f are the densities of the composites, the neat matrix, and the nanoparticles respectively, while V_m and V_f are the volume fraction of the matrix and the nanofiller.

The voids content in nanocomposites were evaluated following equation:

$$V_V = \frac{\rho_{\exp} - \rho_{th}}{\rho_{th}} \tag{S3}$$

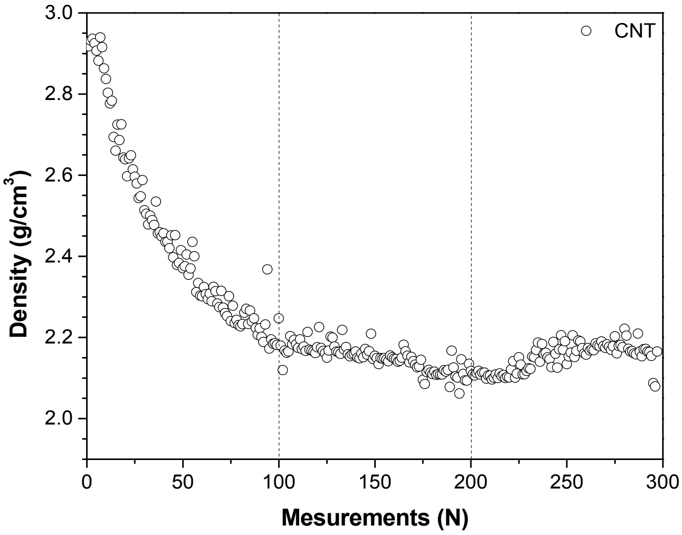

Figure S3. Density of carbon nanotube measured through a Micromeritics®Accupyc 1330 helium pycnometry (23 °C) with 10 cm^3 chamber.

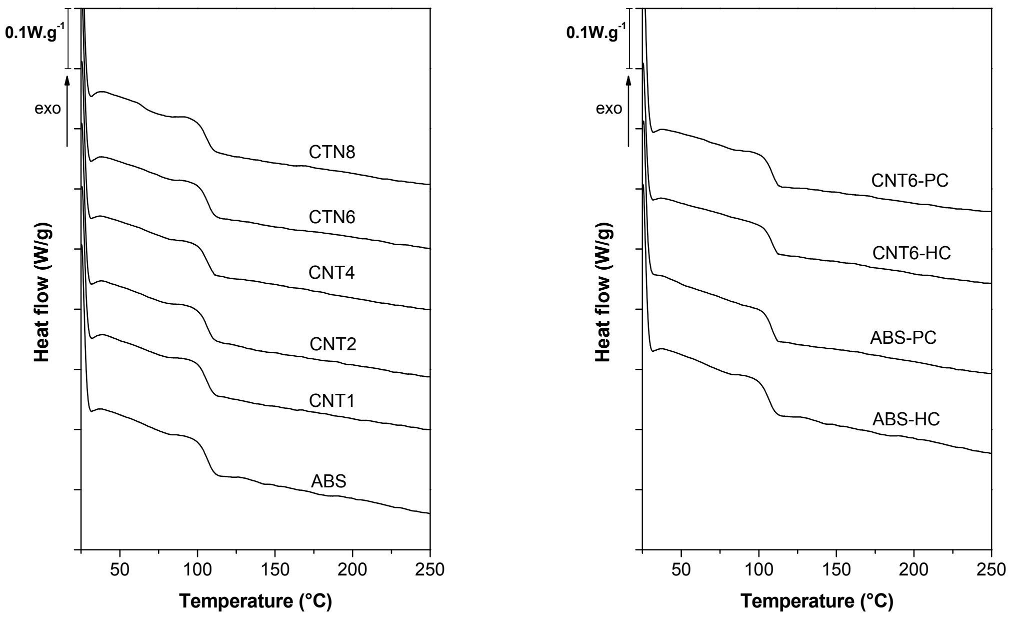

(**a**) (**b**)

Figure S4. DSC thermogram of neat ABS and its ABS/CNT nanocomposites: (**a**) filaments; (**b**) 3D-printed samples.

Table S2. Glass transition temperatures (T_g) of styrene–acrylonitrile phase in ABS and in nanocomposite (from inflection point of DSC thermogram).

Samples	First heating	Cooling	Second heating
		T_g (°C)	
ABS	106.1	102.1	108.0
CNT1	105.8	102.2	108.7
CNT2	105.5	102.9	108.8
CNT4	105.5	102.7	108.8
CNT6	106.7	100.1	109.2
CNT8	105.2	102.7	108.8
ABS-HC	109.0	101.9	108.1
ABS-VC	108.1	101.1	108.3
CNT6-HC	108.5	102.4	109.7
CNT6-VC	108.5	102.9	108.7

3. Fracture of Cross-section of 3D Printed Samples

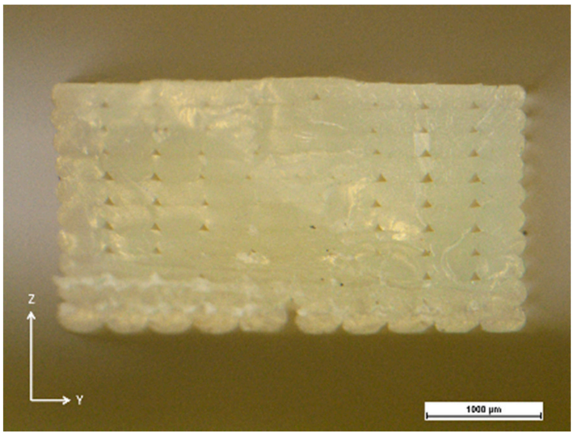

(a)

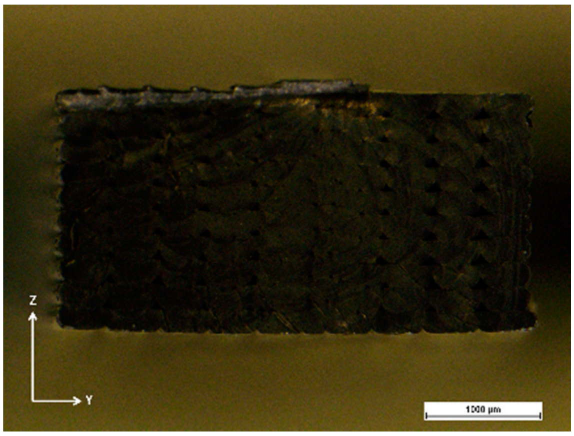

(d)

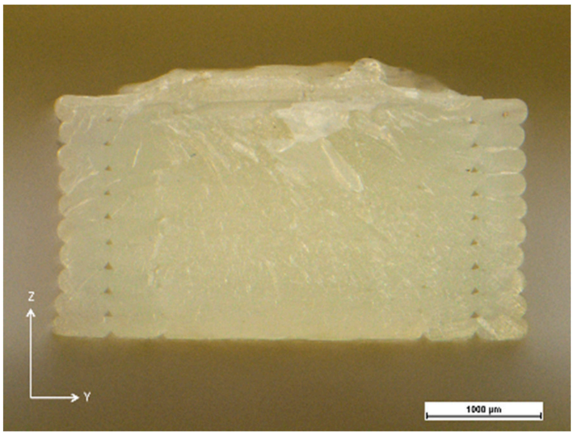

(**b**)

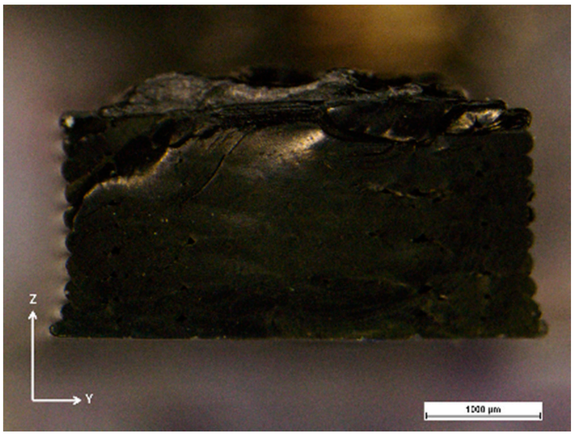

(**e**)

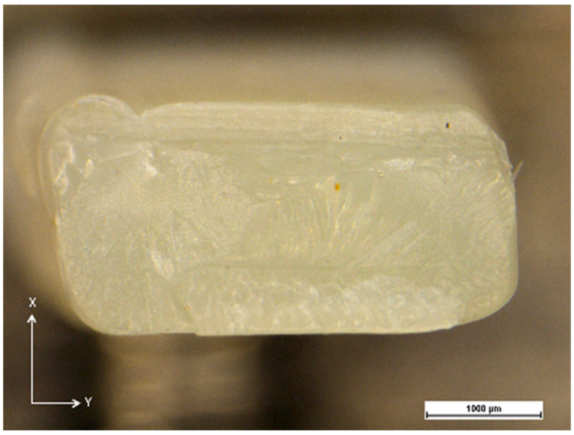

(**c**)

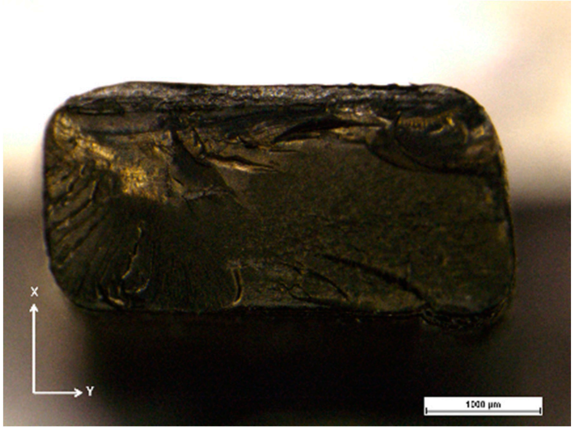

(**f**)

Figure S5. Frozen fracture of cross-section of 3D-printed dumbbells: (**a**) ABS-HC, (**b**) ABS-H45, (**c**) ABS-VC, (**d**) CNT6-HC, (**e**) CNT6-H45 and (**f**) CNT6-VC.

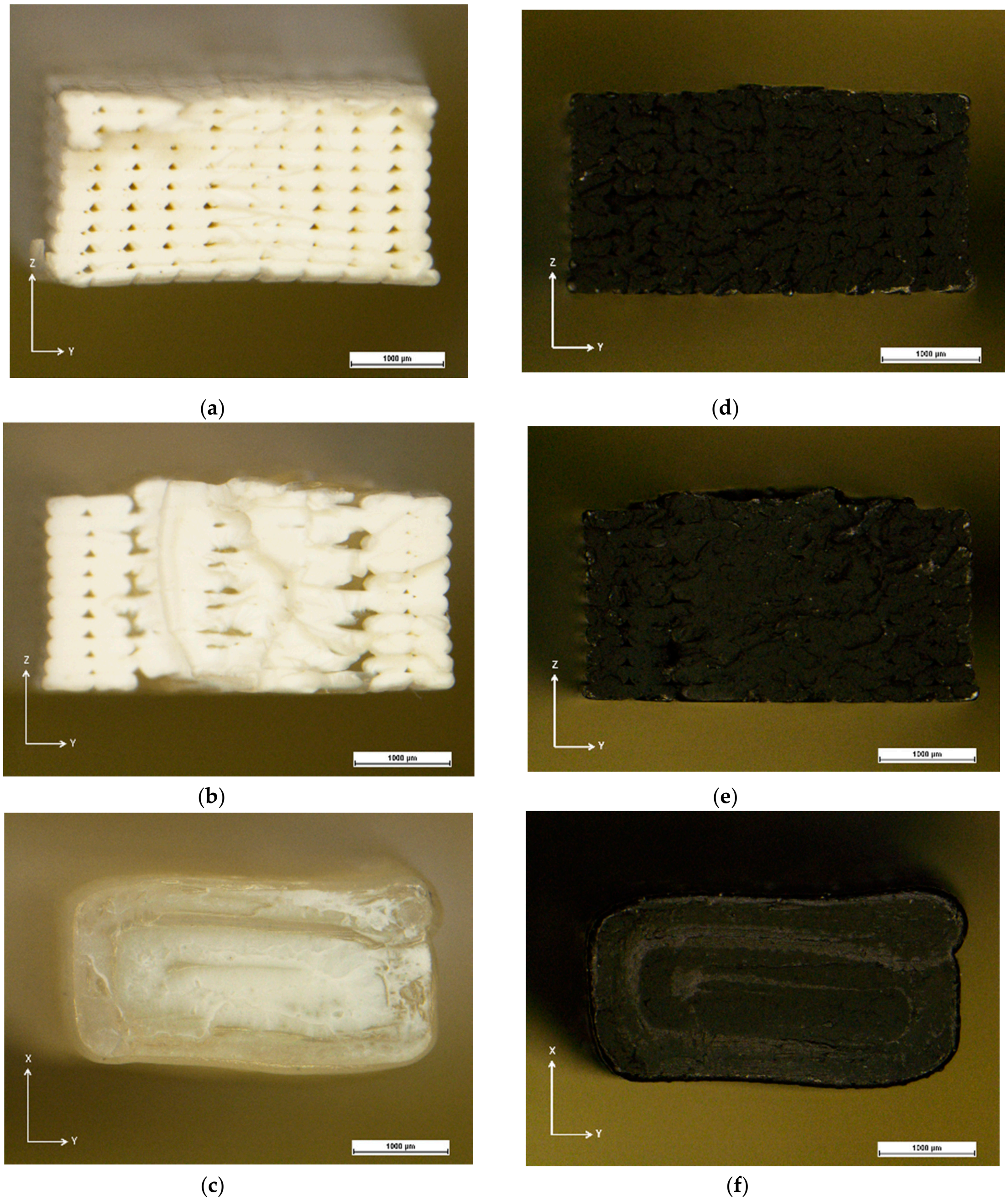

Figure S6. Tensile fracture of cross-section of 3D-printed dumbbells: (**a**) ABS-HC, (**b**) ABS-H45, (**c**) ABS-VC, (**d**) CNT6-HC, (**e**) CNT6-H45 and (**f**) CNT6-VC.

Table S3. Evaluation of orientation factor in microfilament during 3D printing (according Eq. (S4)).

Samples	Freely extruded fiber diameter D_f (µm)*	Deposited microfilament**			Microfilament orientation OF_{microF}
		Width (µm)	Height (µm)	Equivalent diameter D_{microF} (µm)	
ABS	648	420	210	335	3.74
CNT6	515	410	210	331	2.42

* from Table 5.

** dimensions of microfilament were determined from Figures S5a and S5d.

The orientation factor for microfilament production in 3D-printing, OF_{microF}, could be calculated from the relative ratio between the cross-sectional area of freely extruded fiber (S_f), and the cross-sectional area of the microfilament (S_F) according to Equation S4:

$$OF_{microF} = S_f/S_{microF} = (D_f / D_{microF})^2 \qquad (S4)$$

where D_f and D_{microF} are the diameter of fiber and equivalent diameter of microfilament

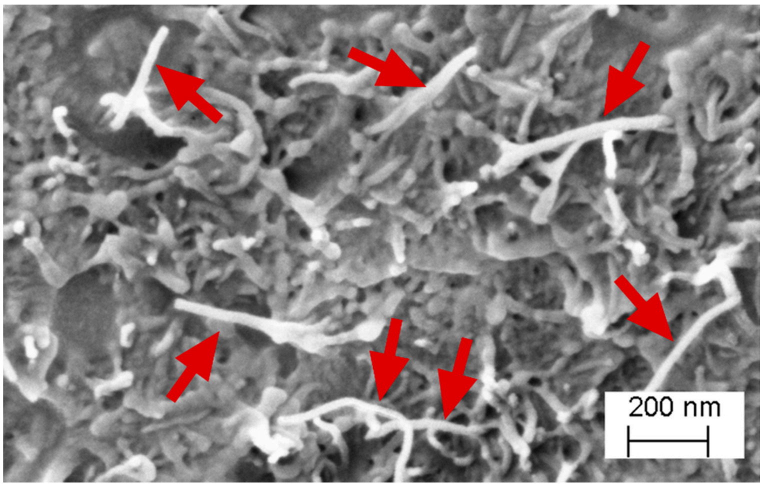

Figure S7. SEM micrographs of 3D-printed dumbbell specimens of CNT6-HC with indicating CNTs (red arrow).

Table S4. Volume resistivity of different kinds of ABS-CNT samples at an applied voltage of 5 V.

CTN content (wt%)	Filament Φ = 1.70 mm (Ω.cm)	3D-printed fiber Φ = 0.50–0.55 mm (Ω.cm)	3D samples (Ω.cm)
2%	/	520 ± 95	Not prepared
4%	10.78 ± 0.25	8.41 ± 1.26	Not prepared
6%	4.12 ± 0.15	2.05 ± 0.09	61.2 ± 7.0 (HC)<br>49.0 ± 4.8 (H45)<br>16.8 ± 2.7 (VC)
8%	1.84 ± 0.15	1.18 ± 0.04	Not prepared

4. Production of Compression Molded Plates from Filaments

Filaments CNT6 and CNT8 with a final diameter of 1.70 mm were selected for the preparation of compression molded plates. Single filaments were accurately aligned in parallel (Figure S8a) and then hot pressed in a Carver Laboratory press at a temperature of 190°C under a pressure of 3.2 MPa for 5 min, in order to produce 50×50×1 mm plate, as shown in Figure S8b.

Different oriented specimens (25×6×1 mm) for electrical measurements were machined from the plates as shown in Figure S8c. The correspondent resistivity measurements are shown in Figure S9.

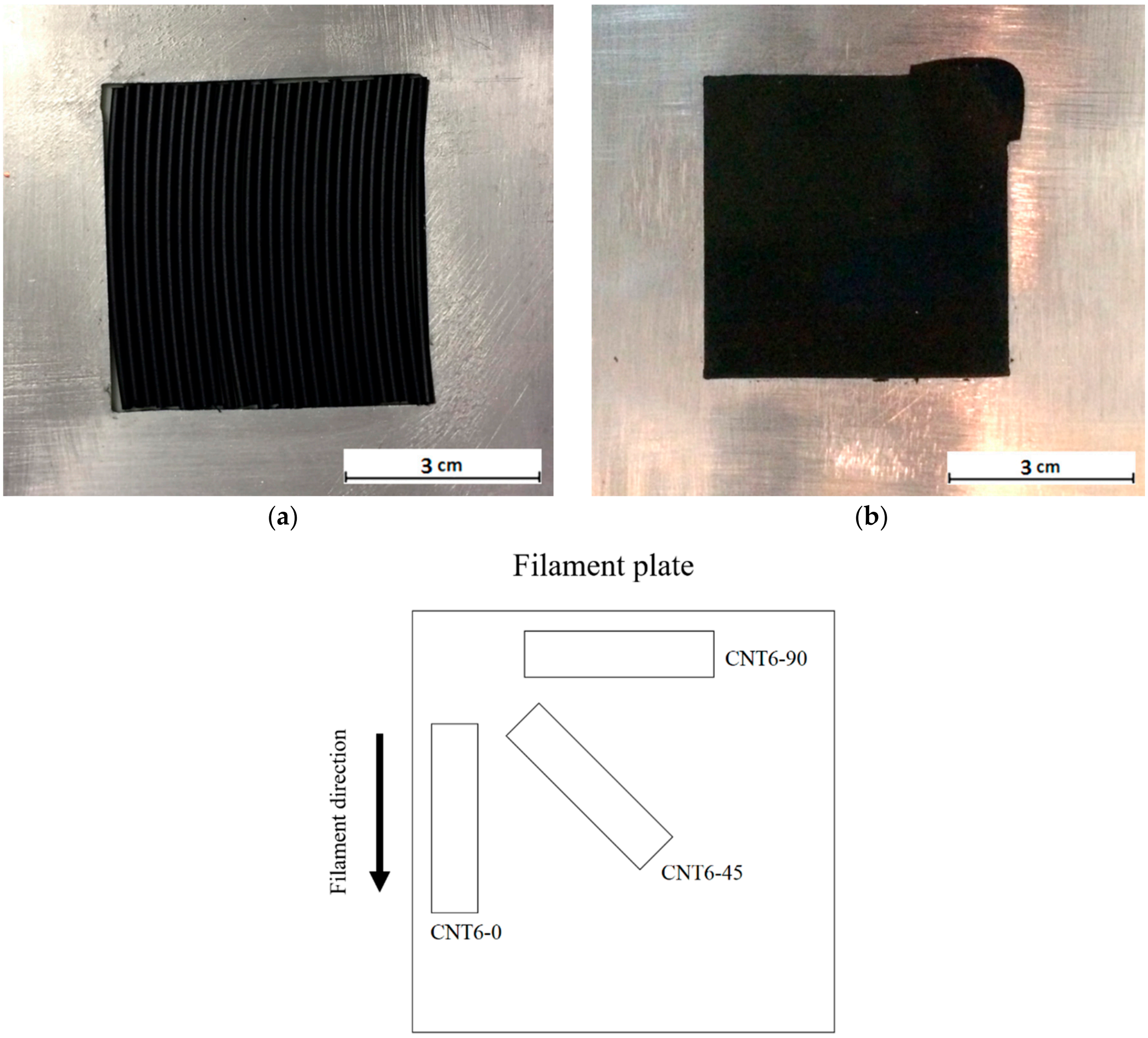

(**c**)

Figure S8. Summary of preparation of filament plate with the mould 50 mm × 50 mm × 1.0 mm starting with filaments at 6 and 8 wt% of CNT: (**a**) before compression, and (**b**) after compression. (c) Schematic of samples at the different angles (0, 45 and 90°) for measuring electrical resistivity (see Figure S9).

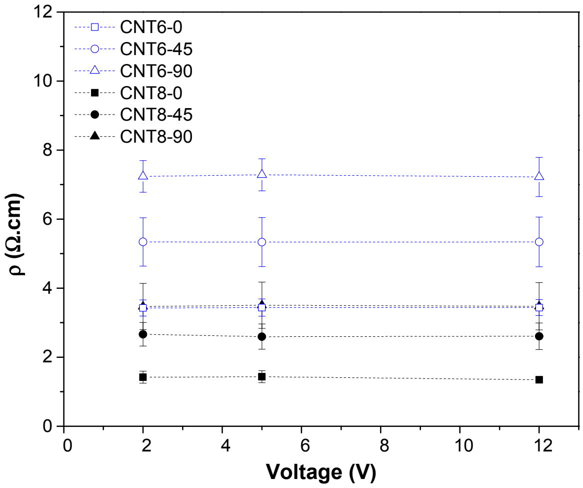

Figure S9. Electrical volume resistivity of ABS 6 wt% and 8 wt% filled nanocomposites of filament plates at different angles (0, 45 and 90°) as a function of the applied voltage.
